# Tailored Mesoporous Silica Nanoparticles and the Chick Chorioallantoic Membrane: A Promising Strategy and Model for Efficient Blood-Brain Barrier Crossing

**DOI:** 10.1021/acsami.5c05429

**Published:** 2025-05-06

**Authors:** Cong-Kai Lin, Yi-Shan Yang, Tsang-Pai Liu, Jiunn-Chang Lin, Sasinan Bupphathong, Fuyuhiko Tamanoi, Yi-Ping Chen

**Affiliations:** a Graduate Institute of Biomedical Materials Tissue Engineering, College of Biomedical Engineering, 548311 Taipei Medical University, Taipei 110, Taiwan; b Department of Neurosurgery, 63474 Taipei Medical University Hospital, Taipei 110, Taiwan; c Department of Surgery, 36897 MacKay Memorial Hospital, Taipei 104, Taiwan; d MacKay Junior College of Medicine, Nursing and Management, New Taipei 252, Taiwan; e Graduate Institute of Nanomedicine and Medical Engineering, College of Biomedical Engineering, 38032 Taipei Medical University, Taipei 110, Taiwan; f Institute for Integrated Cell-Material Sciences, Institute for Advanced Study, 12918 Kyoto University, Kyoto 606-8501, Japan; g International Ph.D. Program in Biomedical Engineering, College of Biomedical Engineering, 38032 Taipei Medical University, Taipei 110, Taiwan

**Keywords:** blood-brain barrier, mesoporous silica nanoparticles, chick chorioallantoic membrane, PEG chain, surface charge, drug delivery

## Abstract

Crossing the blood-brain barrier (BBB) remains a major challenge for brain-targeted drug delivery. Mesoporous silica nanoparticles (MSNs) with tunable size and surface properties are promising vehicles for crossing the BBB. In this study, we explored the potential applications of the chick chorioallantoic membrane (CAM) model in combination with nanotherapeutics. We synthesized ∼25 nm MSNs and RITC-conjugated MSNs (RMSNs) with short PEG chains and varying amounts of positively charged molecules, specifically tertiary amine (polyethylenimine, PEI) or quaternary amine (trimethylammonium, TA), to investigate the positive charge effects on BBB penetration. Strongly positively charged TA-modified RMSNs (s-RMSN@PEG/TA, where s denotes strongly positively charged) effectively crossed the chick embryo BBB, whereas PEI-modified RMSNs did not. Although the weakly positively charged formulation (w-MSN@PEG/TA, where w denotes weakly positively charged) exhibited higher Dox loading capacity and a faster release rate, s-MSN@PEG/TA demonstrated superior BBB penetration and drug permeability. Consistent with chick CAM results, RMSN@PEG/TA also penetrated the BBB in mice. Long-chain PEG-modified RMSN@PEG/TA (RMSN@PEG_(L)_/TA, where L denotes long-chain PEG) showed reduced BBB penetration due to steric hindrance, possibly shielding TA molecules. This study highlights the effectiveness of optimizing short PEG chain density and TA modification for MSN-based BBB crossing without additional biological ligands. Furthermore, the chick CAM model proves to be a valuable alternative to mouse models for assessing BBB crossing of nanoparticles, offering significant research opportunities.

## Graphical abstract

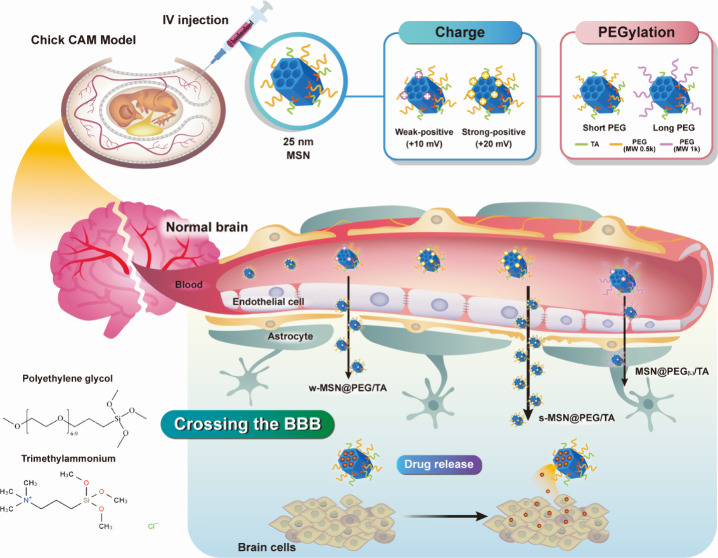

## 1 Introduction

The blood-brain barrier (BBB) serves as a critical interface between neural tissues and circulating blood, functioning as a homeostatic defense mechanism for the brain. Despite the prevalence of clinical trials related to brain diseases, many of these efforts have encountered limited success, primarily due to the impermeability of the BBB to most therapeutic agents.^1,2^ This inherent restriction poses a significant challenge to the delivery of drugs to the brain, allowing only a minimal amount of therapeutic molecules to cross the barrier.^3^ Consequently, overcoming this challenge and developing innovative delivery systems have become urgent priorities in the treatment of brain-related diseases.

Nanoparticle-based drug delivery systems (NDDS) have emerged as a promising approach to enhance drug availability and facilitate the traversal of the BBB, offering new opportunities for treating brain disorders.^4^ Various mechanisms are involved in the transport of nanoparticles (NPs) across the BBB, including passive and active targeting.^5^ Among these, size and surface charge are crucial factors influencing NPs transport across the BBB and their interaction with brain cells.^6,7^ Small-sized NPs are particularly efficient at crossing the BBB due to their ability to pass through the tight junctions between endothelial cells, which act as barriers to substance entry into the brain.^8^ Additionally, smaller NPs exhibit improved diffusion properties, allowing them to pass through the BBB and reach the desired brain tissue.^9,10^ This emphasizes the importance of NPs size as a critical factor in overcoming BBB barriers.

Positively charged NPs may enhance BBB crossing due to the unique properties and charge selectivity of the BBB.^11^ The BBB is composed of tightly packed endothelial cells connected by tight junctions, creating a negative surface charge owing to the presence of glycoproteins and other charged components on the cell surfaces.^12^ As a result, electrostatic interactions between positively charged NPs and endothelial cells can facilitate BBB crossing. Moreover, positively charged NPs can undergo adsorptive-mediated transcytosis, a process in which they adhere to the endothelial cell membrane and are subsequently transported through the cell.^13^ This mechanism allows NPs to bypass the tight junctions between endothelial cells, favoring efficient delivery to the brain. Research has shown that positively charged NPs exhibit approximately 100 times greater permeability across the BBB compared to their neutral counterparts.^7^ Thus, the surface charge effect of NPs is of considerable importance in the design of effective drug delivery systems across the BBB.

When NPs are injected into the bloodstream, they undergo rapid adsorption of serum proteins onto their surfaces, forming a dynamic layer known as the protein corona.^14,15^ This phenomenon is highly relevant in nanomedicine, as the protein corona can significantly influence NP behavior, thereby affecting therapeutic efficacy. The surface properties of NPs play a pivotal role in the formation of protein coronas, directly impacting therapeutic outcomes. Polyethylene glycol (PEG) coating is a common strategy in NDDS to enhance NP performance, including extending circulation time, reducing immune system recognition, enhancing bioavailability, and mitigating the protein corona effect.^16^ However, an excessive PEG chain length can lead to steric hindrance, potentially shielding ligands or molecules on the NP surfaces, which may impede their interaction with BBB receptors or transporters and hinder effective BBB penetration.^17^ Therefore, optimizing the PEG chain length and density is crucial in designing NPs to efficiently penetrate the BBB. Understanding and managing the protein corona effect, particularly concerning PEG coating, is vital for enhancing the success of NDDS in treating brain diseases.

To gain a more comprehensive understanding of BBB function and dysfunction, various in vitro and in vivo models have been employed. In vitro assays, such as transwell assays using cultured rodent or nonhuman mammalian cells, are used to measure the permeability of substances through a monolayer of BBB endothelial cells, allowing researchers to assess BBB tightness.^18,19^ While in vitro models offer precise control of experimental conditions, high throughput, and low ethical concerns, they fall short of fully replicating the complexity of the BBB in living organisms.^20,21^ In contrast, in vivo models, such as those using zebrafish (Danio rerio) or mice, provide valuable insights into physiological relevance, disease states, pathological changes, therapeutic outcomes, and BBB integrity within a living organism.^22–24^ However, these models come with strict ethical concerns, high production costs, and interspecies variability.^25^ Thus, a balanced approach that combines the strengths of both in vitro and in vivo models is crucial for advancing BBB-related research and treatment development.

A 2022 review published in ACS Nano by Liang et al. highlighted the chick chorioallantoic membrane (CAM) model as a valuable bridge between in vitro and in vivo studies.^26^ Their work emphasized the chick CAM’s capacity to accelerate the validation and qualification of NPs for in vivo investigations, showcasing its potential as an alternative in vivo model. Previous reports have indicated that the BBB integrity of the chick CAM matures around embryonic day 12, at which point the tight junctions between endothelial cells strengthen, restricting the passage of various substances similar to the mature BBB of the mammalian brain.^27^ Despite the inherent differences from the human BBB, the chick CAM model offers a cost-effective and efficient alternative to traditional mouse models, providing valuable contributions to specific aspects of BBB research, particularly in drug delivery and high-throughput screening of BBB-penetrating drugs. These findings suggest that the chick CAM model could serve as a reliable tool for studying BBB permeability and substance passage, making it a promising alternative to conventional mouse models in BBB research.

Mesoporous silica nanoparticles (MSNs) offer a highly tunable platform for the controlled delivery of therapeutics, owing to their adjustable pore size, large surface area, and versatile surface functionalization. These characteristics make MSNs particularly attractive for central nervous system (CNS) drug delivery, where precise targeting and protection of labile therapeutic agents are essential. In recent years, MSN-based drug delivery systems have been developed for the treatment of neurodegenerative diseases such as Alzheimer’s disease (AD), Parkinson’s disease (PD), and amyotrophic lateral sclerosis (ALS). For instance, González et al. designed an MSN-based nanocarrier coloaded with leptin and pioglitazone, which was able to cross the BBB and demonstrated neuroprotective effects in an ALS mouse model by reducing inflammation and oxidative stress.^28^ Similarly, other studies have employed MSNs to deliver therapeutic agents, such as curcumin or fluorescent probes of SZIs, into the brains of AD mice, aiming to successfully modulate neuroinflammation or facilitate amyloid-beta imaging in vivo.^29,30^ Collectively, these studies underscore the promise of MSNs as versatile nanocarriers for addressing the complex challenges of neurodegenerative disease therapy. Additionally, Brinker et al. recently demonstrated the use of tailored MSNs by integrating the CAM model to directly observe their in vivo behavior, including selective leukemia cell targeting, vascular margination, circulation dynamics, stability, and reduced endothelial binding after intravenous injection.^31–34^ The CAM model enables high-resolution, real-time imaging of MSN interactions with tissues and cells in a live system, providing a cost-effective and accessible platform for assessing nanocarrier fate in vivo.

In this study, we developed 25 nm MSNs functionalized with either positively charged tertiary amine (polyethylenimine, PEI) or quaternary amine (trimethylammonium, TA) molecules, along with polyethylene glycol (PEG), to investigate the effects of surface charge and PEG chain length on cellular internalization, BBB penetration, and drug delivery efficacy (Scheme 1). Our findings revealed that RITC-conjugated MSNs (RMSNs) functionalized with short PEG (M.W. 459–591) and TA (denoted RMSN@PEG/TA) showed dose-dependent cellular uptake and no cytotoxicity, making them favorable for therapeutic applications. In contrast, RMSNs modified with short PEG and PEI (denoted RMSN@PEG/PEI) exhibited significantly higher cytotoxicity, likely due to the inherent toxicity of PEI, despite better cellular uptake. RMSN@PEG/TA demonstrated improved BBB permeability without compromising BBB integrity. In comparison, RMSN@PEG/PEI failed to achieve significant BBB crossing, highlighting that TA was crucial for facilitating MSN penetration across the BBB. Brain imaging and silicon content analysis revealed higher accumulation of s-RMSN@PEG/TA (s indicated stronger positive charge) in chick brain tissues, indicating superior BBB penetration compared to w-RMSN@PEG/TA (w indicated weaker positive charge). Additionally, Dox-loaded MSN@PEG/TA showed dose-dependent cytotoxicity in U87 glioma cells, confirming their therapeutic potential. Dox-loaded w-MSN@PEG/TA (Dox@w-MSN@PEG/TA) exhibited efficient drug loading and a pH-responsive release profile, with accelerated release under acidic conditions (pH 5.5), characteristic of tumor environments. Despite the lower Dox drug loading capacity of s-MSN@PEG/TA, its enhanced surface charge facilitated more efficient BBB crossing and delivery of Dox to brain tissues, achieving significantly higher brain accumulation compared to free Dox and Dox@w-MSN@PEG/TA. The BBB penetration ability was compromised when TA molecules were hindered by long PEG chains (M.W. One k) in RMSN surface modification (denoted RMSN@PEG_(L)_/TA, L stands for long-chain). Hence, we demonstrated that the MSN with optimized short PEG density can retain the advantages of PEG while preserving the functionality of TA, facilitating effective BBB crossing. This straightforward design strategy based on TA modification and the optimization of short PEG chains, enables MSNs to cross the BBB without the need for additional BBB-penetrating ligands.

**Scheme 1:**
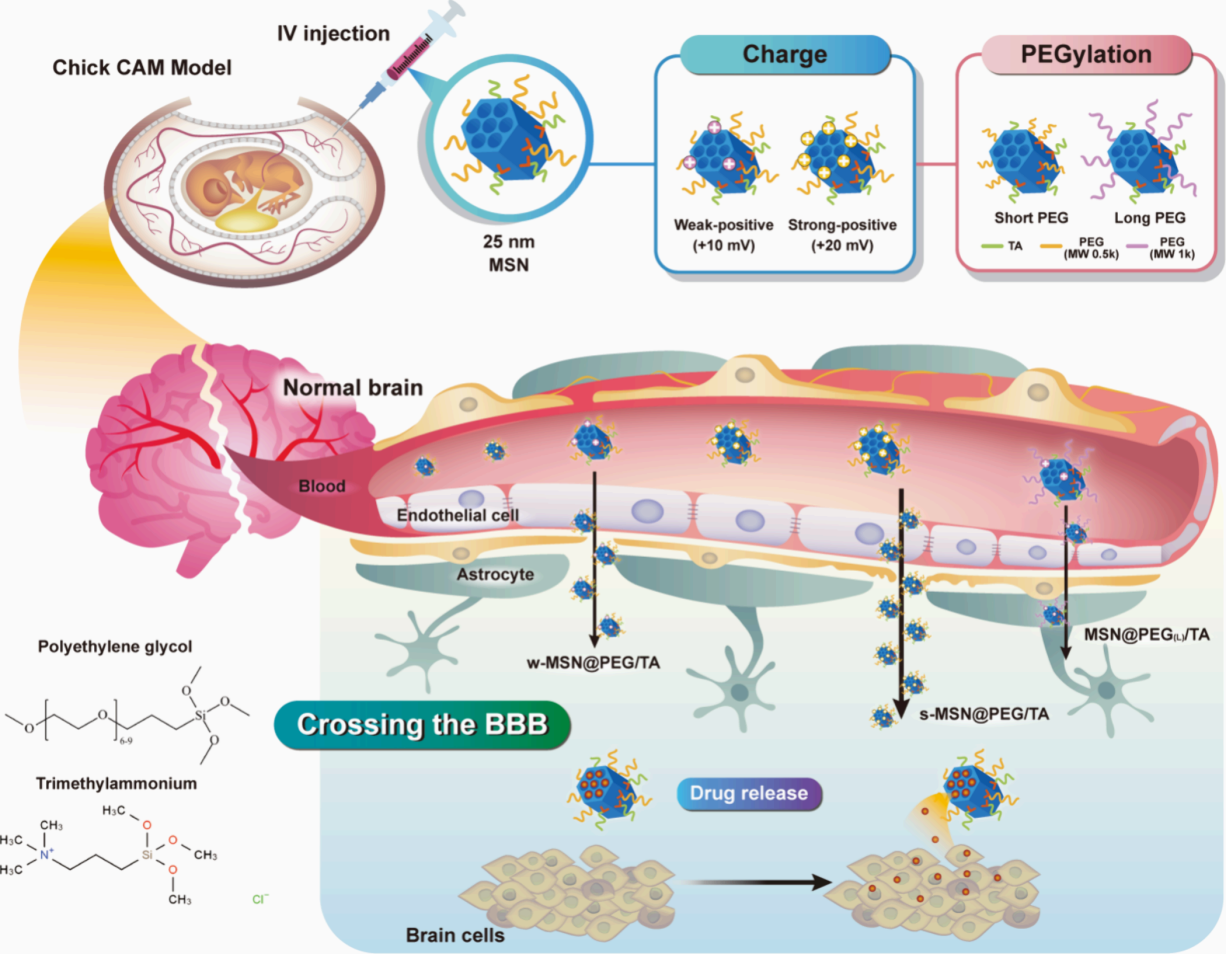
Schematic Representation of 25 nm MSNs Functionalized with Positively Charged Tertiary Amine (PEI) or Quaternary Amine (TA) and Polyethylene Glycol (PEG) to Examine the Impact of Surface Charge and PEG Chain Length on Cellular Uptake, BBB Penetration, and Drug Delivery Efficacy

Additionally, although the chick CAM model is well-established in tumor and angiogenesis research,^35,36^ its application in BBB research remains limited yet holds significant potential. In this study, the chick CAM model effectively mimicked the intact BBB of mammalian systems, with trypan blue staining confirming BBB maturity on embryonic day 15. The consistency between chick CAM and mouse models supports the use of chick CAM as a reliable and cost-effective alternative in vivo model for assessing BBB permeability.

## 2 Materials and Methods

### 2.1 Chemicals and Reagents

All reagents were obtained from commercial suppliers and used without further purification. Doxorubicin (Dox), sodium bicarbonate (≥99.7%), Rhodamine B isothiocyanate (RITC), and Fluorescein Isothiocyanate-Dextran (FITC-Dextran, M.W. 40 kDa) were purchased from Sigma-Aldrich (Germany). Ammonium hydroxide solution (28.0–30.0% in water), cetyltrimethylammonium bromide (CTAB, 99%+), tetraethyl orthosilicate (TEOS, 98%), and 3-Aminopropyltrimethoxysilane (APTMS, 95%) were sourced from Acros Organics (Germany). 2-[Methoxy­(polyethyleneoxy)­6–9propyl]­trimethoxysilane (PEG-silane, M.W. 459–591 g/mol), 2-[Methoxy­(polyethyleneoxy)­21–24propyl]­trimethoxysilane (PEG_(L)_-silane, M.W. One k g/mol, where L stands for long chain), N-trimethoxysilylpropyl-N,N,N-trimethylammonium chloride (TMAC-silane, 50% in methanol, M.W. 257.83), and trimethoxysilylpropyl-modified (polyethyleneimine) (PEI-Silane, 50%, M.W. 1500–1800) were obtained from Gelest (Morrisville, PA, USA). Trypan blue (0.4%) was purchased from Thermo Fisher Scientific (Waltham, MA, USA). Fetal bovine serum (FBS) and penicillin were obtained from HyClone Laboratories (Logan, UT, USA). Cell Counting Kit-8 (CCK-8) was acquired from Dojindo Laboratories (Japan).

### 2.2 Preparation of Mesoporous Silica Nanoparticles (MSNs) and RITC-Conjugated MSNs (RMSNs)

Typically, 0.29 g of CTAB was dissolved in 150 mL of a 0.128 M aqueous ammonium hydroxide solution. The resulting mixture was sealed with parafilm and incubated in a water bath at 60 °C for 15 min with continuous stirring. Subsequently, 2 mL of 0.88 M TEOS predissolved in ethanol was gradually added dropwise to the solution under continuous stirring. For the synthesis of RITC-conjugated MSNs (RMSNs), 8 mg of RITC was dissolved in 5 mL of 99.5% ethanol and stirred for 15 min. Subsequently, 10 μL of APTMS was added to the solution, and the mixture was stirred under dark conditions at room temperature for 24 h to produce preconjugated RITC-APTMS before adding 2 mL of 0.88 M TEOS solution.

After 1 h, a mixture of PEG-silane or PEG_(L)_-silane (1.07 mmol) and TA-silane (0.54 mmol for weak positive charge and 2.2 mmol for strong positive charge) or PEI-silane (0.83 μmol for weaker positive charge and 5.57 μmol for stronger positive charge) was introduced to the solution. The mixture was gently stirred for 1 h and then the solution was aged and concentrated to one-third of its original volume at 60 °C for 24 h. The obtained NPs were then subjected to hydrothermal treatment at 70 °C for 24 h, followed by a second treatment at 90 °C for an additional 24 h. The surfactants were removed by hydrochloric acid extraction at 60 °C, and the NPs were washed twice with ethanol using a cross-flow filtration system. Finally, template-removed RMSNs with a diameter of 25 nm, carrying different amounts of positively charged ligands of polyethyleneimine (PEI) or a quaternary amine group (TA-silane) were obtained, including stronger positive charges (denoted s-RMSN@PEG/PEI and s-RMSN@PEG/TA) and weaker positive charges (denoted w-RMSN@PEG/PEI and w-RMSN@PEG/TA), and stored in 95% ethanol.

### 2.3 Characterization of MSNs

The morphology and mesoporous structural features of the RMSNs were characterized using transmission electron microscopy (TEM, HITACHI HT7700, Tokyo, Japan) operated at an acceleration voltage of 60 kV. TEM images were analyzed with Scanpro software to determine the size distribution of the MSNs. The surface area and pore size of the NPs were measured using the Brunauer–Emmett–Teller (BET) and Barrett–Joyner–Halenda (BJH) calculation methods based on nitrogen adsorption–desorption isotherms (Micromeritics ASAP 2020, USA). The mean hydrodynamic size of the NPs was determined using dynamic light scattering (DLS, Zetasizer Nano ZS90, Malvern Instruments, Worcestershire, UK). Zeta potential measurements were conducted to assess the surface charge of the NPs through electrophoretic mobility, also using the Zetasizer Nano ZS90. Elemental analysis for nitrogen, carbon, sulfur, and hydrogen (NCSH) content was performed using an Elementar Vario EL cube instrument (Germany), providing the detailed elemental composition of the RMSNs. The chemical functional groups present in the various MSNs were identified using FTIR spectroscopy (Nicolet iS10 FTIR Spectrometer, Thermo Fisher Scientific).

### 2.4 Cell Culture

The human glioma U87-MG cell line was maintained in Dulbecco’s modified Eagle’s medium (DMEM) supplemented with 10% fetal bovine serum (FBS), 100 units/mL penicillin, 100 μg/mL streptomycin, 1 mM sodium pyruvate, and 1 mM nonessential amino acids. The cells were incubated at 37 °C in a humidified atmosphere containing 5% CO2. Subculturing was performed when the cells reached approximately 80% confluence.

### 2.5 Cellular Uptake

U87-MG cells were seeded in 6-well plates at a density of 2 × 10^5^ cells per well and cultured overnight. Subsequently, RMSNs were added to each well at the indicated concentrations (250, 500, 750, and 1000 μg/mL) in DMEM complete medium and incubated for an additional 24 h. The cellular uptake of RMSNs in U87-MG cells was imaged under an inverted fluorescence microscope (OPTIKA IM-3FL4, Italy) and quantified using flow cytometry by detecting the RITC fluorescence signals, respectively.

### 2.6 Cytotoxicity Assay

U87-MG cells were seeded in 96-well plates at a density of 1 × 10^4^ cells per well in DMEM complete medium and incubated for 24 h. Following different RMSNs treatments at various concentrations (1, 2.5, 5, 10, and 20 μg/μL) for an additional 24 h, cells were treated with Cell Counting Kit-8 (CCK-8, Dojindo, Japan) for an additional 4 h. The absorbance of each well was then measured at 450 nm using a microplate reader (Thermo Fisher Scientific, Waltham, MA, USA) to assess cell viability.

Preparation of Dox-Loaded MSNs: 20 mg of MSN@PEG/TA (without RITC conjugation) was dispersed in 400 μL of 0.1 M NaHCO_3_ and incubated for 30 min, followed by washing twice with double-distilled (dd) H_2_O. The washed MSN@PEG/TA was redispersed in 1 mL ddH_2_O and added to 1.427 mg Dox dissolved in 2 mL ddH_2_O. The mixture was incubated in the dark at 25 °C for 1 h with gentle agitation and centrifuged at 3500 rpm using a Vivaspin-20-centrifugal-concentrator, followed by washing with ddH_2_O to eliminate any remaining unloaded Dox. The concentration of unloaded Dox was determined by measuring its fluorescence intensity with excitation and emission wavelengths set at 480 and 560 nm, respectively

The Dox loading efficiency (%) and loading amount (%) of NPs were calculated using the following equations:

$$\mathrm{loading}\;\mathrm{efficiency}\;(\%)=\frac{\mathrm{weight}\;\mathrm{of}\;\mathrm{Dox}(\mathrm{Dox}@\mathrm{MSN}@\mathrm{PEG}/\mathrm{TA})}{\mathrm{weight}\;\mathrm{of}\;\mathrm{total}\;\mathrm{Dox}}\times 100\%$$

$$\mathrm{loading}\;\mathrm{amount}\;(\%)=\frac{\mathrm{weight}\;\mathrm{of}\;\mathrm{Dox}(\mathrm{Dox}@\mathrm{MSN}@\mathrm{PEG}/\mathrm{TA})}{\mathrm{weight}\;\mathrm{of}\;\mathrm{Dox}@\mathrm{MSN}@\mathrm{PEG}/\mathrm{TA}}\times 100\%$$

### 2.7 In Vitro Dox Release Profile

0.5 mL of Dox and Dox-loaded MSNs suspension at a concentration of 0.2 mg/mL were enclosed in a dialysis membrane with a molecular weight cutoff of 12–14 kDa. The dialysis membrane was then immersed in 1.7 mL of phosphate-buffered saline (PBS) at either pH 7.4 or pH 5.5 and maintained at 37 °C with gentle shaking. At predetermined time intervals, 1 mL samples of the released Dox were collected and analyzed using a fluorescence spectrometer (JASCO FP-8500, UK) at an excitation wavelength of 480 nm and an emission wavelength of 560 nm. The concentration of released Dox was determined by referencing a previously established calibration curve for Dox in PBS at both pH 7.4 and pH 5.5.

### 2.8 Western Blot Analysis

Following NP treatment, cells were lysed using RIPA buffer supplemented with protease inhibitors and incubated on ice for 2 h. The lysates were then centrifuged to collect the supernatant containing the extracted proteins. The protein concentration in the supernatant was determined using the Bradford protein assay. Subsequently, 20 μg of protein extract were separated by electrophoresis on a 10% SDS-PAGE gel and then transferred to a polyvinylidene fluoride (PVDF) membrane (EMD Millipore, Merck). The PVDF membrane was blocked in PBS containing 0.1% Tween 20 (PBS-T) with 5% (w/v) BSA for 1 h and then washed three times with PBS-T. Primary antibodies against p-p38 (sc-17852-R, Santa Cruz Biotechnology, USA), HMGB1 (ab227168, abcam, UK), and α-tubulin (sc-5286, Santa Cruz Biotechnology, USA) were incubated with the PVDF membrane at 4 °C overnight with gentle rocking. After washing three times with TBS-T, the membrane was further incubated with an HRP-conjugated secondary immunoglobulin G antibody (Santa Cruz Biotechnology, USA) in TBS-T containing 5% (w/v) bovine serum albumin (BSA) for 2 h at room temperature. Finally, signals corresponding to the protein bands were visualized using an enhanced chemiluminescent substrate kit (Amersham Pharmacia Biotech, GE Healthcare, Bucks, UK) according to the manufacturer’s protocol.

### 2.9 BBB Model of Chick CAM and BBB Integrity Measurement

Fertilized eggs were purchased from JD-SPF Biotech (Taiwan) and placed in an incubator at 37 °C with 70% humidity. To assess BBB integrity in chick brains, trypan blue staining was used. On embryonic day 13 or 16, a small hole with a diameter of 5 mm was created in the eggshell for intravenous injection. Subsequently, 50 μL of 0.4% trypan blue solution was injected into the chick CAM using a 31G needle for 24 h. Finally, chick embryos were euthanized and perfused with normal saline containing 0.15% EDTA, and the brains and major organs (heart, liver, spleen, lung, and kidney) were then harvested. Photographs of the trypan blue staining distribution in the tissues were captured. Additionally, the collected organs were fixed in 4% formaldehyde and embedded in optimal cutting temperature (OCT) for subsequent frozen sectioning. The distribution of trypan blue was imaged under a fluorescence microscope by capturing the red fluorescence signals emitted by trypan blue with an excitation wavelength of 633 nm.

### 2.10 Brain Imaging Observation

The chick CAM was euthanized 24 h after the intravenous injection of either RMSNs (at concentrations equivalent to Dox loaded MSNs), Dox alone (0.03 mg/egg), or Dox loaded MSNs (at concentrations equivalent to Dox alone) on embryonic day 15 and the brain was excised. Images of RMSNs and Dox distribution were obtained with an IVIS imaging system (IVIS Lumina III XRMS), capturing the fluorescence signals of RITC at an excitation wavelength of 580 nm and an emission wavelength of 620 nm, or Dox an excitation wavelength of 480 nm and an emission wavelength of 590 nm. The radiant efficiency, defined as fluorescence intensity/area/time, was analyzed using the IVIS imaging software with the region of interest (ROI) tool. To visualize blood vessels, 50 μL of fluorescein isothiocyanate-dextran (FITC-dextran) at 2.5 mg/mL was intravenously injected 24 h post-treatment (embryonic day 16). After injection, the skull and dura mater of the brain were carefully removed, and brain images were photographed using a two-photon high-resolution microscope (Zeiss LSM 7 MP, Germany).

### 2.11 ICP-MS/MS Analysis

The Si content in the brain of chick CAM was quantitatively determined using ICP-MS/MS (Agilent 8900 ICP-MS/MS, USA). Briefly, fresh brains were weighed and subsequently subjected to digestion using a 1600 W microwave (95 °C for 20 min, 180 °C for 15 min, and 200 °C for 15 min) in a 1:1:1 mixture of H_2_O/HF/HNO_3_. The resulting digested samples were analyzed by ICP-MS/MS. Si concentration was determined based on a calibration curve prepared with Si standards in a concentration range of 0–200 μg/L. The Si content was reported as nanograms of Si per milligram of brain tissue.

### 2.12 Tissue Distribution of RMSNs and Dox

After 24 h of treatment, chick embryos were humanely sacrificed, and their brains were collected for frozen sectioning. The distribution of RMSNs and Dox released from Dox-loaded MSNs was assessed using confocal microscopy (Leica Stellaris 8 Confocal Microscope, Germany). Red fluorescence signals from RITC (excitation: 580 nm; emission: 620 nm) and Dox (excitation: 480 nm; emission: 590 nm) were captured. DAPI staining was performed to visualize cell nuclei. The percentage of Dox-positive cells was quantified using TissueQuest software (TissueGnostics, Vienna, Austria), which measures the fluorescence intensity of Dox relative to DAPI.

### 2.13 In Vivo Dox Permeability

Collected brains were homogenized using Precellys Homogenizers (Bertin Technologies, France). Tissue samples were homogenized with acidified methanol (50% methanol in 0.3 N HCl) and centrifuged at 24,000 × g for 10 min at 4 °C. The fluorescence intensity of the resulting supernatant was measured using a fluorescence spectrometer (JASCO FP-8500, UK) with excitation and emission wavelengths set at 480 and 560 nm, respectively. The concentration of Dox in the brain was determined using a calibration curve established with Dox standards in a concentration range of 0–1000 ng/L. The drug permeability was calculated using the formula shown below:

$$\mathrm{drug}\;\mathrm{permeability}(\%)=\frac{\mathrm{measured}\;\mathrm{Dox}\;(\mathrm{mg})}{\mathrm{Dox}@\mathrm{NPs}\;(1\;\mathrm{mg})}\times 100\%$$

### 2.14 Brain Distribution of RMSNs in Mice

Male BALB/c mice, aged 12 to 14 weeks, were purchased from BioLASCO Experimental Animal Center (BioLASCO, Taipei, Taiwan) and housed under specific pathogen-free (SPF) conditions. All procedures were conducted in accordance with the guidelines of the Laboratory Animal Center of Taipei Medical University. The mice were administered an intravenous injection of NPs at a dosage of 200 mg/kg body weight. After 24 h of treatment, the distribution of s-RMSN@PEG/PEI, s-RMSN@PEG/TA, and s-RMSN@PEG_(L)_/TA in the brain was visualized using an IVIS imaging system (IVIS Lumina III XRMS), capturing the RITC fluorescence signals with an excitation wavelength of 580 nm and an emission wavelength of 620 nm.

### 2.15 Statistical Analysis

Statistical analysis was performed using GraphPad Prism 6 software (GraphPad Software, Inc., San Diego, CA, USA). All experiments were independently conducted at least three times, and the results are expressed as mean ± standard deviation (SD). Statistical significance between different groups was compared using Student’s *t* test. A p-value of <0.05 was considered statistically significant, with significance levels denoted as follows: *p < 0.05, **p < 0.01, ***p < 0.001, and ****p < 0.0001.

## 3 Results

### 3.1 Preparation and Characterization of Various Types of MSNs

PEGylation has emerged as a promising approach to enhance the dispersity and stability of NPs in physiological environments. Additionally, it extends the retention time of NPs in the bloodstream and mitigates the serum protein corona effect. Positively charged NPs offer advantages such as improved cellular uptake and enhanced ability to cross the BBB. In this study, we synthesized MSNs with a small size of 25 nm and focused on evaluating the impact of surface properties and charge effects on BBB penetration. Four types of 25 nm MSNs were obtained by tailoring the PEG (M.W. 459–591) content and incorporating varying amounts of two positively charged molecules: polyethyleneimine (PEI, M.W. 1500–1800) and a quaternary amine group (TA-silane, M.W. 257.83). Based on their surface characteristics, these MSNs were categorized into strong (designated as s-MSN@PEG/PEI and s-MSN@PEG/TA) and weak (designated as w-MSN@PEG/PEI and w-MSN@PEG/TA) groups (Figure 1a).

**Figure 1:**
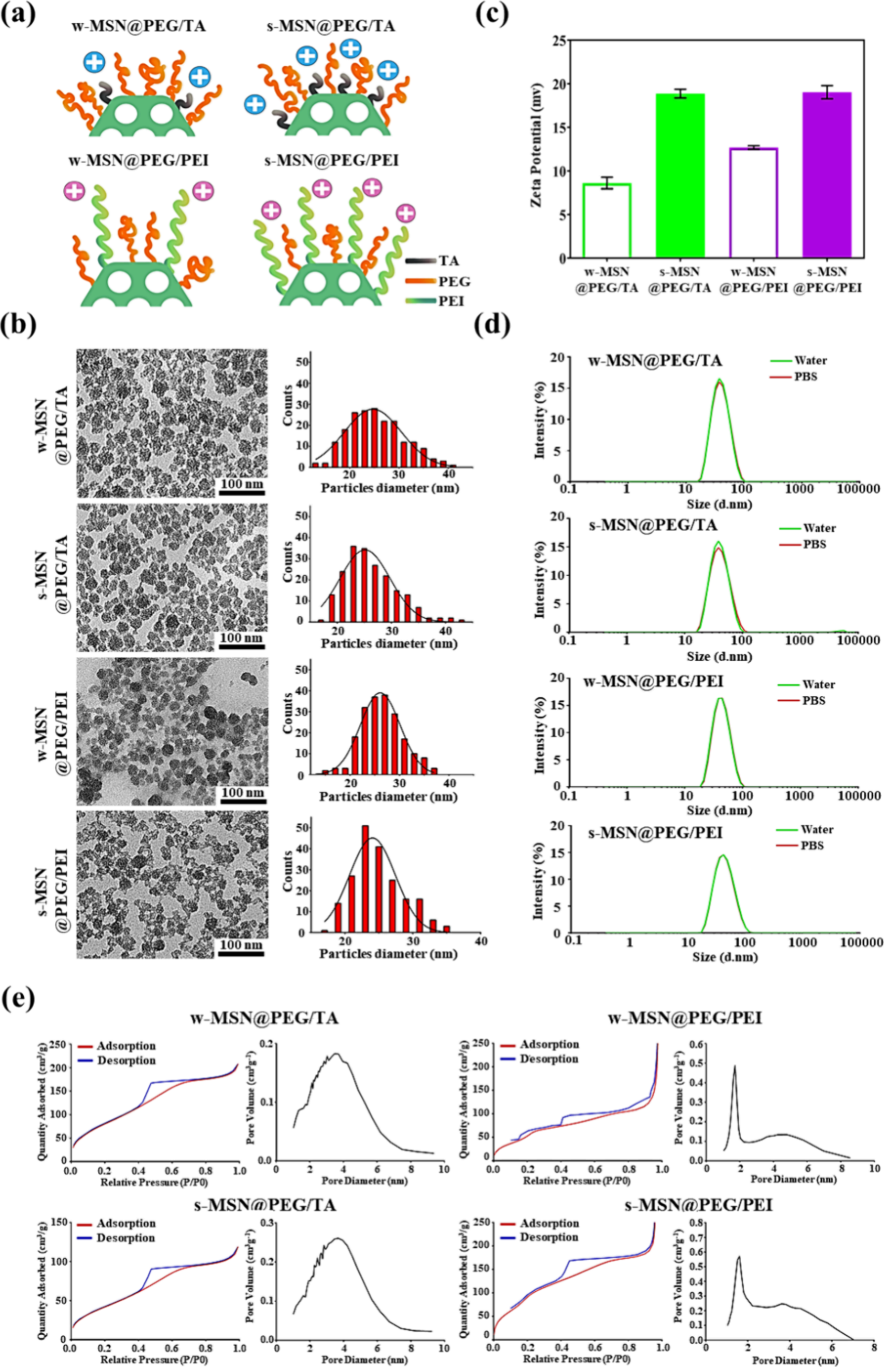
Physical and chemical properties of various positively charged MSNs. (a) Schematic illustration depicting the structural features of different MSNs. (b) TEM images and size distribution histograms of MSNs. Scale bar: 100 nm. (c) Zeta potential measurements of MSNs. (d) DLS analysis showing the size distribution of MSNs in ddH_2_O and PBS. (e) Nitrogen adsorption–desorption isotherms and the corresponding pore size distribution plot of MSNs.

As depicted in Figure 1b, all MSNs exhibited a uniform morphology and well-defined mesoporous structure, as observed in the transmission electron microscopy (TEM) images. Zeta potential measurements (Figure 1c) revealed that the surface charges of w-MSN@PEG/TA and w-MSN@PEG/PEI were 9.1 ± 1.08 mV and 13.6 ± 1.70 mV, respectively. In contrast, s-MSN@PEG/TA and s-MSN@PEG/PEI exhibited stronger positive charges of 18.1 ± 1.10 mV and 19.1 ± 0.75 mV, respectively. The increased positive surface charges of s-MSN@PEG/TA and s-MSN@PEG/PEI were attributed to the higher ratios of positively charged TA or PEI molecules conjugated to the MSNs. Dynamic light scattering (DLS) measurements indicated that the MSNs were well-suspended with particle sizes ranging from 38 to 44 nm in both water and PBS (Figure 1d). Elemental analysis provided the atomic percentages of carbon (C), hydrogen (H), and nitrogen (N) for the different MSNs, as summarized in Table S1. Notably, s-MSN@PEG/TA, with a higher proportion of TA modification, exhibited a greater nitrogen content compared to w-MSN@PEG/TA. Similar trends were observed for s-MSN@PEG/PEI and w-MSN@PEG/PEI with varying ratios of PEG and PEI. The typical characteristics of mesoporous materials, such as a type IV isotherm with a hysteresis loop, surface area (*S*_BET_), and pore diameter (DBJH), were measured using nitrogen adsorption–desorption isotherms (Figure 1e). All the basic characterization of various MSNs is summarized in Table 1. Ultimately, we successfully synthesized 25 nm PEGylated MSNs with distinct surface charges and positively charged ligands (PEI and TA). This study investigates the charge effects of these various MSNs on BBB penetration, demonstrating their potential as carriers for drug delivery.

**Table 1:** Basic Characterization of the Various MSNs

		**size (nm)** ^a^				
**sample**	**average size from TEM (nm)**	**water (PDI)** ^b^	**PBS (PDI)** ^b^	**ζ-potential (mv)** ^c^	***S***_**BET**_ **(m^2^/g)** ^d^	***D***_**BJH**_ **(nm)** ^e^
w-MSN@PEG/TA	24.67 ± 1.43	38.3 ± 0.77 (0.15)	39.0 ± 0.71 (0.14)	9.1 ± 1.08	364.5	2.4
s-MSN@PEG/TA	25.13 ± 2.18	43.0 ± 0.06 (0.13)	37.6 ± 0.15 (0.01)	18.1 ± 1.10	362.9	2.3
w-MSN@PEG/PEI	26.09 ± 1.70	42.2 ± 0.34 (0.20)	42.7 ± 0.49 (0.20)	13.6 ± 1.70	242.5	2.2
s-MSN@PEG/PEI	24.05 ± 4.42	43.5 ± 0.32 (0.17)	43.9 ± 0.12 (0.19)	19.1 ± 0.75	398.6	2.1

a Measured by DLS.

b Polydispersity index.

c Measured by a zeta potential analyzer.

d *S*_BET_: surface area calculated from data using the BET equation.

e *D*_BJH_: pore diameter assigned from the maximum on the BJH pore size distribution.

### 3.2 Assessment of the BBB Integrity in the Brain of Chick CAM

To evaluate the ability of various MSNs to cross the BBB, subsequent experiments were conducted using the chick CAM model. The experimental design, illustrated in Figure 2a, outlines the timeline and procedure for establishing the chick CAM model, as detailed in the experimental section. For qualitative analysis, brain imaging and histological staining of tissue sections will be used to visually assess the distribution of MSNs and Dox. Quantitative analysis will involve tissue homogenization followed by data quantification, providing precise measurements of the distribution and concentration of MSNs and Dox in various tissues, thereby offering a comprehensive understanding of their in vivo biodistribution.

**Figure 2:**
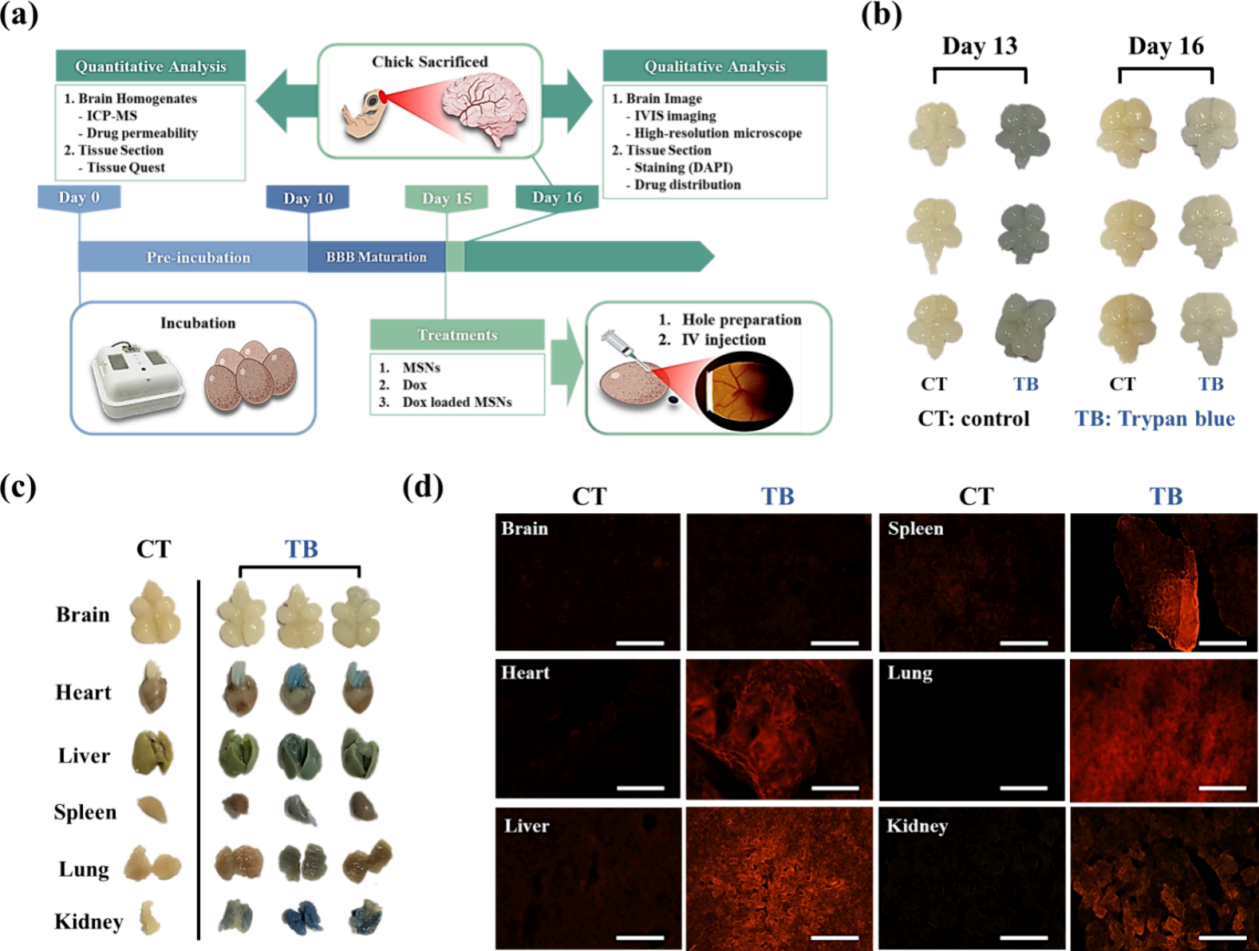
Trypan blue staining of BBB in the brain of chick CAM. (a) Schematic illustration of the chick CAM model used for BBB studies. (b) Analysis of BBB maturation on embryonic day 13 (embryonic immature) and 16 (embryonic mature) using trypan blue staining. (c) Photograph and (d) fluorescence images (shown in red). Images depicting trypan blue staining in chick CAM brains and major organs on embryonic day 16, following injection on embryonic day 15. Scale bar: 100 nm.

To assess BBB integrity in chick brains, fertilized eggs were preincubated for either 12 days (representing embryonic immaturity) or 15 days (representing embryonic maturity). A small window, approximately 1 cm in diameter, was cut into the eggshell for intravenous injection of NPs. At 24 h postinjection (embryonic day 13 or 16), the chick embryos were humanely sacrificed, and major organs were excised for both quantitative and qualitative analyses. Evidence from MRI observations strongly suggests that the structural development of the chick embryo brain reaches full maturity after embryonic day 13.^27^ To assess the integrity of the BBB at different stages of chick CAM maturation, trypan blue dye was used as a marker to validate BBB permeability. Trypan blue, due to its hydrophilicity (from sulfonic acid groups) and large molecular size (MW = 960.8 g/mol, 1150 Da), has limited capability to pass through a fully developed and intact BBB.^37,38^ When the BBB is not fully developed, trypan blue can leak from the bloodstream into brain tissue, resulting in visible brain staining.^39,40^ This phenomenon serves as an indicator of BBB integrity. In this study, BBB integrity in the chick CAM was assessed by intravenous injection of trypan blue (0.4%) on embryonic days 12 (embryonic immature) and 15 (embryonic mature). The brains were excised 1 day postinjection for examination. Photographs in Figure 2b revealed noticeable trypan blue staining on embryonic day 13, whereas brain samples from embryonic day 16 showed no staining, confirming that the BBB was fully developed in the chick embryo by embryonic day 15.

To further examine trypan blue-specific permeability in major organs (including the heart, liver, spleen, lungs, and kidneys) relative to the brain, these organs were excised from trypan blue-injected day 16 chick CAM embryos and imaged. Figure 2c demonstrated that trypan blue staining was evident in all organs except for the brain and the control CAM, indicating that trypan blue could not traverse the BBB to stain the brain tissue. Subsequently, the collected organs were processed for frozen tissue sectioning and imaged under a fluorescence microscope to capture the red fluorescence signal of trypan blue (λex: 633 nm). Figure 2d showed that there was no red fluorescence signal in the control group organs. In contrast, the trypan blue-injected chick CAM organs exhibited a robust red fluorescence signal in major organs but notably absent in the brain, consistent with the results shown in Figure 2c. The inability of trypan blue to penetrate brain tissue highlights the potential of the chick CAM model as a valuable model for studying BBB integrity and permeability.

### 3.3 RMSN@PEG/TA Traverses the BBB without Causing Disruption and Facilitates Penetration in the Live Chick CAM

To track the NP distribution and cellular internalization in vitro and in vivo, various RITC- conjugated MSNs (RMSNs) were prepared. Cellular internalization of various RMSNs was assessed in U87 glial cells treated with different concentrations (250–1000 μg/mL) for 24 h using flow cytometry analysis. Figure 3a revealed a dose-dependent increase in cellular uptake for s-RMSN@PEG/TA, with approximately 80% uptake at a concentration of 1000 μg/mL. In contrast, w-RMSN@PEG/TA exhibited significantly lower uptake, around 10%, even at higher concentrations. Both s-RMSN@PEG/PEI and w-RMSN@PEG/PEI consistently demonstrated nearly 100% cellular uptake across all concentrations tested. Figure S1 showed fluorescence imaging of cellular uptake of RMSNs at various concentrations, consistent with the results in Figure 3a. To evaluate NP-induced cytotoxicity, the U87 glial cell line was treated with varying NP concentrations (20–400 μg/mL) for 24 h, and cytotoxicity was measured using the CCK-8 assay (Figure 3b). The results showed no cytotoxicity in the RMSN@PEG/TA-treated groups, whereas the w-RMSN@PEG/PEI-treated groups exhibited significant cell death. Additionally, s-RMSN@PEG/PEI-treated groups showed dose-dependent cytotoxicity, indicating that PEI molecules induce more pronounced cytotoxic effects compared to TA molecules.

**Figure 3:**
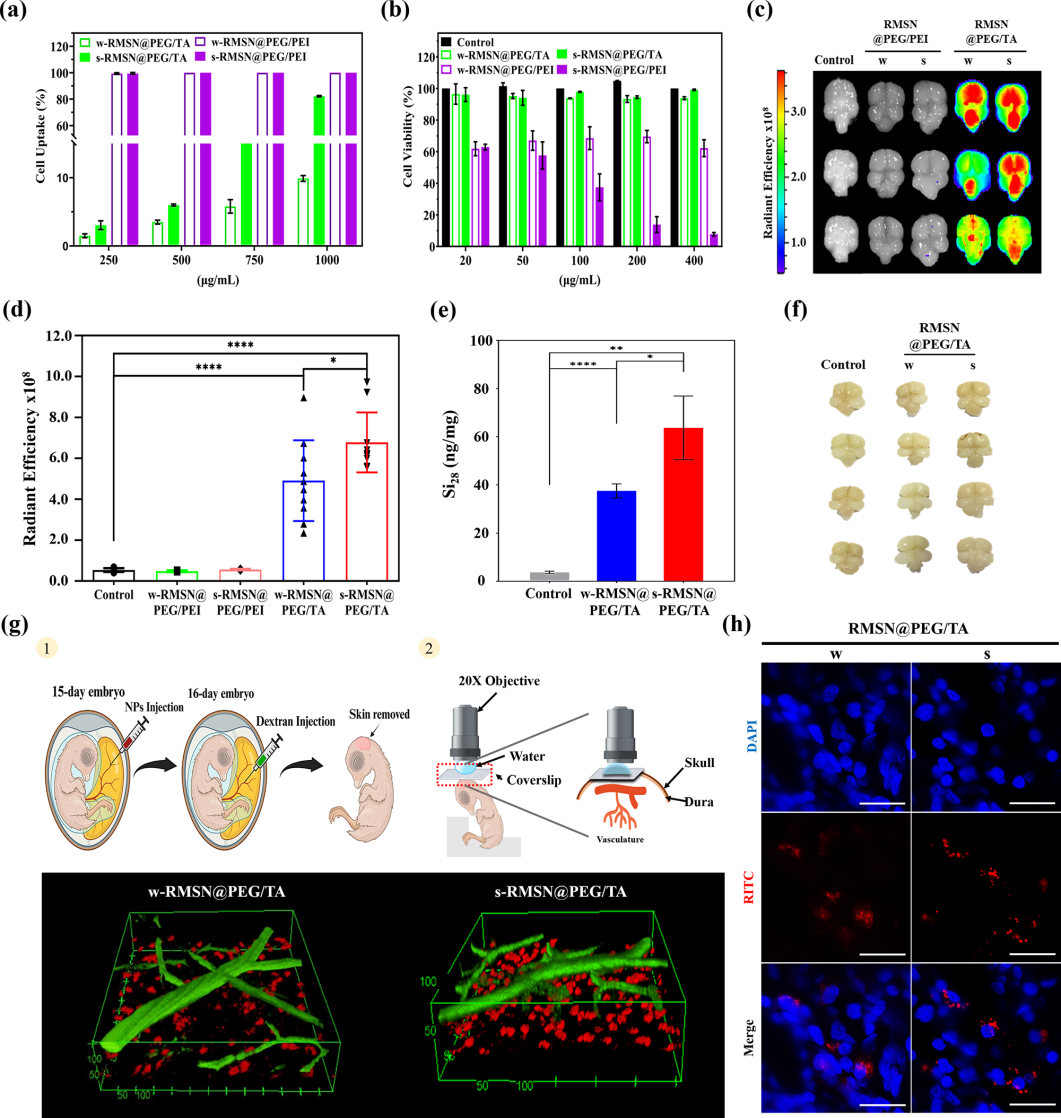
Evaluation of various RMSNs on cellular internalization, cytotoxicity in U87 cells, and their efficacy in crossing the BBB in the chick CAM. U87 cells were treated with various MSNs at indicated concentrations. (a) Cellular uptake assessed using flow cytometry. (b) Cytotoxicity analyzed by the CCK-8 assay. The chick CAM received intravenous injection of various RMSNs at a dose of 1 mg/egg on embryonic day 15 and were sacrificed 24 h after treatment. (c) IVIS imaging of brain distribution. (d) Radiant efficiency of RMSNs in chick embryonic brains. *N* = 10. (e) Quantitative analysis of Si content in chick brains using ICP-MS. (f) BBB integrity analysis of chick CAM 24 h after treatment with RMSN@PEG/TA. (g) Biodistribution of RMSNs in live embryonic chick brain. Schematic illustration of the chick calvarial window model under a two-photon microscope. 3D reconstructed images from two-photon microscopy showing RMSN@PEG/TA (red) and FITC-dextran (green) distribution. (h) Confocal microscopy images of brain sections with RMSN@PEG/TA (red) and DAPI-stained nuclei (blue). Scale bar: 20 μm. Data are presented as mean ± SD. Statistical significance was analyzed using Student’s *t* test (*p < 0.05, **p < 0.01, ***p < 0.001, ****p < 0.0001).

Next, we explored the in vivo penetration efficiency of various RMSNs across the BBB. Chick CAM models with intact BBB at embryonic day 15 were randomly assigned to five groups (control, s-RMSN@PEG/PEI, w-RMSN@PEG/PEI, w-RMSN@PEG/TA, and s-RMSN@PEG/TA; N = 10 per group) and then intravenously injected with different RMSNs (1 mg/egg). Brains were harvested 24 h post-treatment, and RMSNs distribution was visualized using the IVIS system by capturing the RITC signal (Figure 3c). The RMSN@PEG/TA-treated groups exhibited significantly stronger fluorescence intensity signals compared to the control (nontreated) and RMSN@PEG/PEI-treated groups (Figure 3d). Notably, the RMSN@PEG/PEI-treated groups showed almost no fluorescence intensities, indicating limited BBB penetration. These findings clearly indicated that RMSN@PEG/TA effectively crosses the BBB, while RMSN@PEG/PEI did not, suggesting the importance of surface properties in determining the ability of RMSNs to traverse the BBB. Despite both types of RMSNs possessing positive surface charges, the PEG/TA surface conferred superior BBB penetration compared to the PEG/PEI surface.

Given the higher IVIS fluorescence intensity observed for s-RMSN@PEG/TA relative to w-RMSN@PEG/TA, we further assessed the silicon content in the brain using ICP-MS analysis to quantify the efficiency of RMSNs crossing the BBB. As shown in Figure 3e, the silicon content in the s-RMSN@PEG/TA-treated groups was nearly 1.6 times greater than that in the w-RMSN@PEG/TA-treated groups. To evaluate the structural integrity of the chick CAM BBB following RMSN@PEG/TA treatment, trypan blue staining was performed (Figure 3f). The absence of visible trypan blue staining suggested that RMSN@PEG/TA successfully traversed the BBB without causing disruption. These results indicate that the positively charged TA functionalization of RMSNs enhances BBB penetration without compromising BBB integrity. The study proposes that increased positive surface charge facilitates BBB penetration of RMSN@PEG/TA. However, the biological pathways enabling RMSN@PEG/TA to cross the BBB remain unclear and do not appear to involve direct BBB disruption. Further research is needed to elucidate the underlying mechanisms.

To gain more insight into the ability of RMSN@PEG/TA to cross the BBB, high-resolution imaging was conducted using a two-photon microscope in the chick calvarial window model, as depicted in Figure 3g. On embryonic day 15, after intravenous injection of RMSN@PEG/TA (1 mg/egg), a calvarial window was created in the chick CAM to access the brain 24 h post-treatment. FITC-dextran (2.5 mg/mL), used to stain blood vessels, was injected into the live chick CAM, and brain images were captured using the two-photon microscope. The results showed that RMSN@PEG/TA (red, RITC) was able to leak from blood vessels (green, FITC-dextran). This observation clearly demonstrates that RMSN@PEG/TA is capable of crossing the BBB in the live state of chick CAM brains. Owing to its significant and higher distribution, s-RMSN@PEG/TA exhibited greater efficacy in crossing the BBB compared to w-RMSN@PEG/TA, consistent with previous observations in Figure 3d and Figure 3e.

To further confirm the distribution of NPs within the chick CAM brain, frozen brain tissue sections were prepared and imaged with RMSN@PEG/TA (red, RITC), and nuclei (blue, DAPI) using a confocal microscope (Figure 3h). The images demonstrated that both s-RMSN@PEG/TA and w-RMSN@PEG/TA could penetrate the BBB and were subsequently internalized by cells in the chick CAM brain. Figure S2 presented 3D reconstructed confocal microscopy images of brain sections. The above results suggest that MSNs with a stronger positive charge are more effective in enhancing BBB penetration than those with a weaker positive charge counterpart.

### 3.4 MSN@PEG/TA Promotes the Delivery of Dox into U87 Cells with Slow Release and Enhances Dox Transport Across the BBB in the Chick CAM

NP-based drug delivery has emerged as a promising therapeutic approach. In this study, the small molecule drug doxorubicin (Dox), known for its intrinsic red fluorescence, was chosen for investigation. The loading strategy is based on the electrostatic interactions between the positively charged Dox and negatively charged NPs. The loading efficacy and the amount of Dox in w-MSN@PEG/TA were approximately 69% and 4.7%, respectively, whereas those in s-MSN@PEG/TA were about 22% and 1.6%, respectively (Figure 4a). Figure 4b illustrates the release profiles of Dox and Dox-loaded MSNs under physiological (pH 7.4) and acidic (pH 5.5) conditions. Both w-MSN@PEG/TA and s-MSN@PEG/TA exhibited a slow cumulative release at both pH levels compared to Dox alone. Notably, at equivalent Dox concentrations, the release rate of Dox from w-MSN@PEG/TA and s-MSN@PEG/TA was faster at pH 5.5 than at pH 7.4, indicating pH-responsive release behavior. Importantly, w-MSN@PEG/TA demonstrated superior drug release at pH 5.5 compared to s-MSN@PEG/TA, which showed the slowest cumulative release at both pH levels.

**Figure 4:**
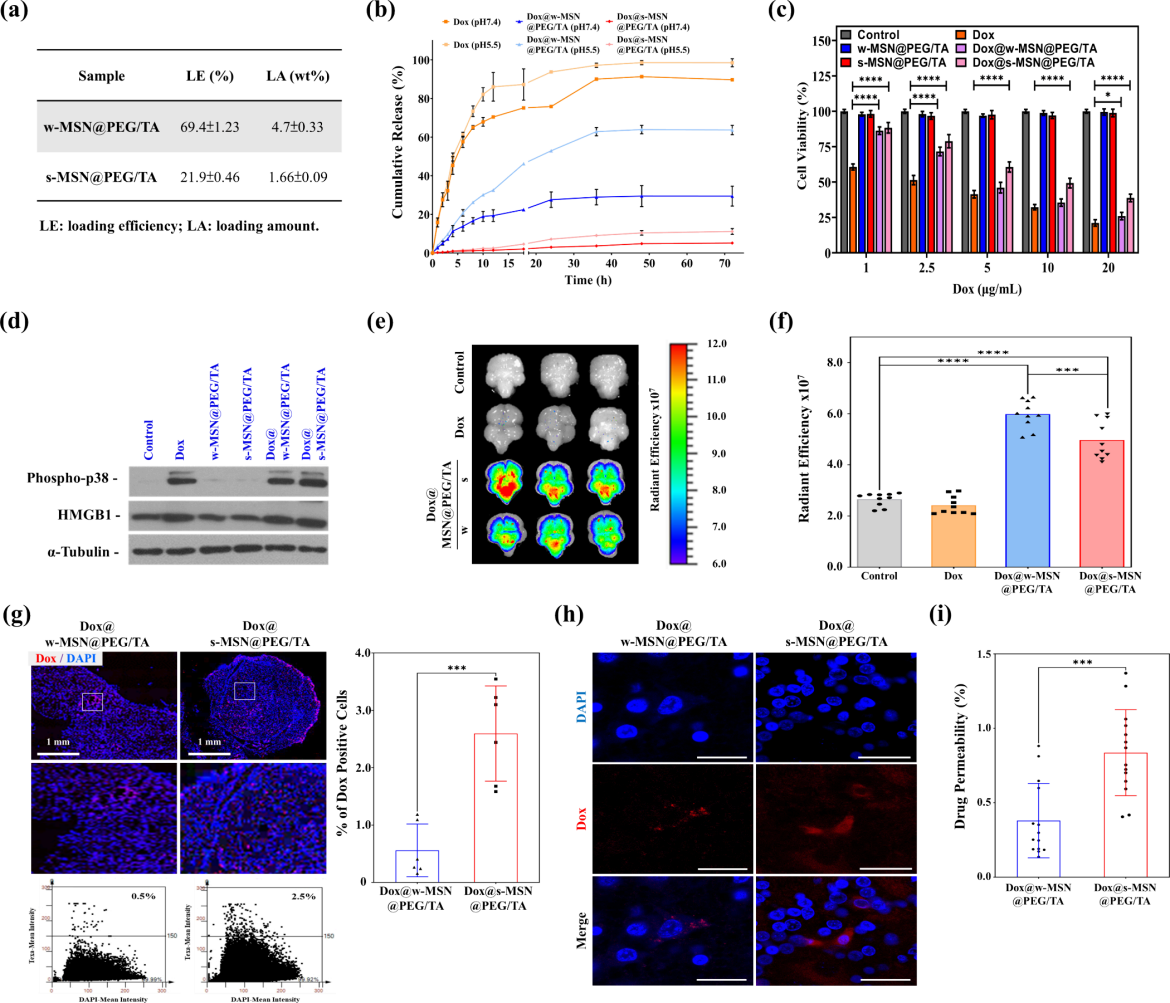
In vitro Dox loading, release, and cytotoxicity in U87 Cells treated with Dox-loaded MSNs and transport of Dox across the BBB in the chick CAM. (a) Loading efficiency and loading amount of Dox-loaded MSNs. (b) Cumulative release profile of Dox from MSMs at pH 7.4 and 5.5. (c) Cytotoxicity analysis of U87 cells treated with Dox alone, MSNs (at equivalent Dox-loaded MSN concentration), and Dox-loaded MSNs (at equivalent Dox alone concentration) across various concentrations (1–20 μg/mL). Cell viability was measured using a CCK-8 assay 24 h after treatment. (d) Western blot analysis showing protein expression levels of Phospho-p38 and HMGB1. α-Tubulin was used as a loading control. (e) IVIS imaging of Dox distribution. (f) Radiant efficiency of Dox in chick embryonic brains. *N* = 10. (g) Quantitative analysis of Dox distribution in brain sections using TissueQuest software. Quantitative analysis of tissue staining and identification of nucleus-positive events were performed using TissueFAXS and TissueQuest software platforms, respectively (red: Dox; blue: DAPI-stained nuclei). (h) Confocal microscopy images of brain sections showing Dox distribution (red) and nuclei (blue, DAPI staining). Scale bar: 20 μm. (i) Drug permeability assay. Quantification of Dox content in brain homogenates using fluorescence spectrophotometry. Data are presented as mean ± SD. Statistical significance was analyzed using Student’s *t* test (*p < 0.05, **p < 0.01, ***p < 0.001, ****p < 0.0001).

After 24 h of treatment, in vitro cytotoxicity assays in U87 cells (Figure 4c) revealed that Dox alone and Dox-loaded MSNs (at concentrations equivalent to Dox alone) induced dose-dependent cell death. MSNs without Dox loading (at concentrations equivalent to those of Dox-loaded MSNs) exhibited no cytotoxicity. Additionally, at treatment concentrations of 5 μg/mL and higher, the toxicity of Dox@w-MSN@PEG/TA was similar to that of Dox alone, whereas Dox@s-MSN@PEG/TA showed significantly lower toxicity than Dox alone. To explore the molecular mechanisms underlying these effects, Western blot analysis was conducted to detect the expression levels of Phospho-p38 (an indicator of Dox-induced free radicals) and HMGB1 (a protein associated with cell apoptosis), confirming that cell death was due to Dox-induced cytotoxicity.^41,42^ As shown in Figure 4d, the Dox-containing groups (Dox, Dox@w-MSN@PEG/TA, and Dox@s-MSN@PEG/TA) exhibited higher expression levels of Phospho-p38 and HMGB1 than the control and MSNs-treated groups without Dox loading, confirming the effective delivery of Dox and subsequent induction of cell death.

Next, Dox delivery to the brain was investigated using MSN@PEG/TA in the chick CAM. Figure 4e illustrates the brain images, where chick CAM was incubated for 15 days, followed by intravenous injection of either Dox alone (0.03 mg/egg), Dox@w-MSN@PEG/TA (at a Dox-equivalent concentration), or Dox@s-MSN@PEG/TA (at a Dox-equivalent concentration). At 24 h postinjection, chick embryo brains were dissected, and red fluorescent signals from Dox (Ex: 480 nm, Em: 590 nm) were analyzed using an IVIS imaging system. As expected, the control and Dox-alone groups showed no signal due to the BBB restrictions, while the Dox@w-MSN@PEG/TA and Dox@s-MSN@PEG/TA-treated groups exhibited significant Dox fluorescence. These results indicated that MSNs effectively facilitated the transport of Dox across the BBB. Quantitative IVIS analysis in Figure 4f revealed a higher red fluorescence intensity in the Dox@w-MSN@PEG/TA-treated groups, possibly due to its superior Dox loading capacity compared to Dox@s-MSN@PEG/TA.

To further confirm the transport and release of Dox via MSNs, frozen brain tissue sections were prepared, and analyzed. Quantitative fluorescence analysis was conducted using Tissue Quest to assess the fluorescence intensity of Dox relative to DAPI staining (Figure 4g). Results indicated that Dox was transported more efficiently across the BBB in the Dox@s-MSN@PEG/TA-treated groups compared to the Dox@w-MSN@PEG/TA-treated groups. Subsequently, the Dox distribution was observed under a confocal microscope (Figure 4h). Numerous red spots were observed in the Dox@w-MSN@PEG/TA- and Dox@s-MSN@PEG/TA-treated groups, suggesting successful Dox transport across the BBB. Nuclear localization was confirmed using DAPI staining (shown in blue), and the overlay of the Dox signal (shown in red) with blue nuclear staining confirmed the intracellular distribution of Dox, providing evidence of effective drug release within the cells. To quantify the Dox content in the brain and compare drug penetration efficiencies between the Dox@w-MSN@PEG/TA- and Dox@s-MSN@PEG/TA-treated groups, brain homogenates were analyzed using spectrofluorometry. As shown in Figure 4i, the drug permeability rate of w-MSN@PEG/TA was approximately 0.25%, whereas that of s-MSN@PEG/TA was approximately 0.75%, which is three times higher than that of w-MSN@PEG/TA. The consistent results between Figure 4g and Figure 4i suggest that Dox is more effectively transported across the BBB when using s-MSN@PEG/TA than when using w-MSN@PEG/TA. Although s-MSN@PEG/TA had a lower Dox loading capacity than w-MSN@PEG/TA, its superior transport and cellular uptake efficiency facilitated more efficient BBB crossing and significantly enhanced drug delivery into cells. Given that the mechanism of action of Dox involves entry into the nucleus to induce cell death, these results support the successful development of MSNs capable of efficiently carrying drugs across the BBB and enabling drug release at the desired cellular targets.

### 3.5 Impact of Long PEG Chains on TA Molecules: Steric Hindrance and Reduced BBB Penetration Efficiency

Long-chain PEG coatings are known to shield NP surface ligands or molecules through steric hindrance, potentially hindering their interaction with BBB receptors or transporters and thereby impeding effective BBB penetration.^17^ To investigate the role of TA molecules on MSNs and their influence on BBB penetration, we replaced the shorter PEG-silane (M.W. 459–591) used in MSN@PEG/TA with long-chain PEG-silane (M.W. One k, designated as PEG_(L)_-silane). Through this modification, w-MSN@PEG_(L)_/TA and s-MSN@PEG_(L)_/TA were synthesized and their ability to penetrate the BBB was compared with that of MSN@PEG/TA using both the chick CAM and mouse models (Figure 5a). As shown in Figure 5b, TEM images reveal that MSN@PEG_(L)_/TA possesses a uniform morphology and a well-defined mesoporous structure. DLS analysis indicated that the average particle sizes of MSN@PEG_(L)_/TA in water and PBS, ranging from 46 to 54 nm, were larger than those of MSN@PEG/TA, demonstrating the impact of the longer PEG chains on overall particle size (Figure 5c and Table 2). BET pore size analysis (Figure 5d and Table 2) showed that the pore sizes of w-MSN@PEG_(L)_/TA and s-MSN@PEG_(L)_/TA were comparable to those of MSN@PEG/TA, suggesting that the long-chain PEG modification did not interfere with the TA surface modification. X-ray diffraction (XRD) patterns exhibited a broad (100) peak, suggesting short-range ordering in the structure of the MSNs (Figure 5e).^43^ The XRD patterns of MSN@PEG/TA and MSN@PEG_(L)_/TA displayed typical diffraction peaks at 2θ values of 2.142°, corresponding to the hexagonal mesoporous silica structure similar to bare MSNs. This indicates that the surface modification did not compromise the structural integrity of the MSNs. As shown in Figure S5, all MSNs exhibited Si–OH (950 cm^–1^) and Si–O–Si (1000–1200 cm^–1^) stretching bands, indicating well-preserved silica frameworks. A distinct C–H stretching band near 2880 cm^–1^confirmed the successful attachment of PEG in all PEG-modified MSNs, while enhanced absorption in the 1550–1650 cm^–1^range in all TA-modified MSNs indicated the incorporation of quaternary ammonium groups. Overall, these FTIR results clearly confirm the effective dual functionalization of MSNs with both PEG- and TA-silane. TGA profiles showed three distinct weight loss stages: the first stage (below 40 °C–200 °C) corresponded to the evaporation of physically adsorbed water; the second stage (200 °C–600 °C) was attributed to the decomposition of PEG/TA; and the final stage (600 °C–800 °C) represented further degradation of PEG/TA. As shown in Figure 5f and Table S2, the weight loss (200 °C–600 °C) for w-MSN@PEG/TA and s-MSN@PEG/TA was 27.4% and 21.1%, respectively, after accounting for the baseline weight loss of bare MSN (6.5%). In contrast, the weight loss for w-MSN@PEG_(L)_/TA and s-MSN@PEG_(L)_/TA was 8.7% and 10.9%, respectively. This reduced weight loss for MSN@PEG_(L)_/TA compared to MSN@PEG/TA can be attributed to the steric hindrance of the long-chain PEG-silane, which limits the accessibility of PEG_(L)_/TA molecules for conjugation.

**Figure 5:**
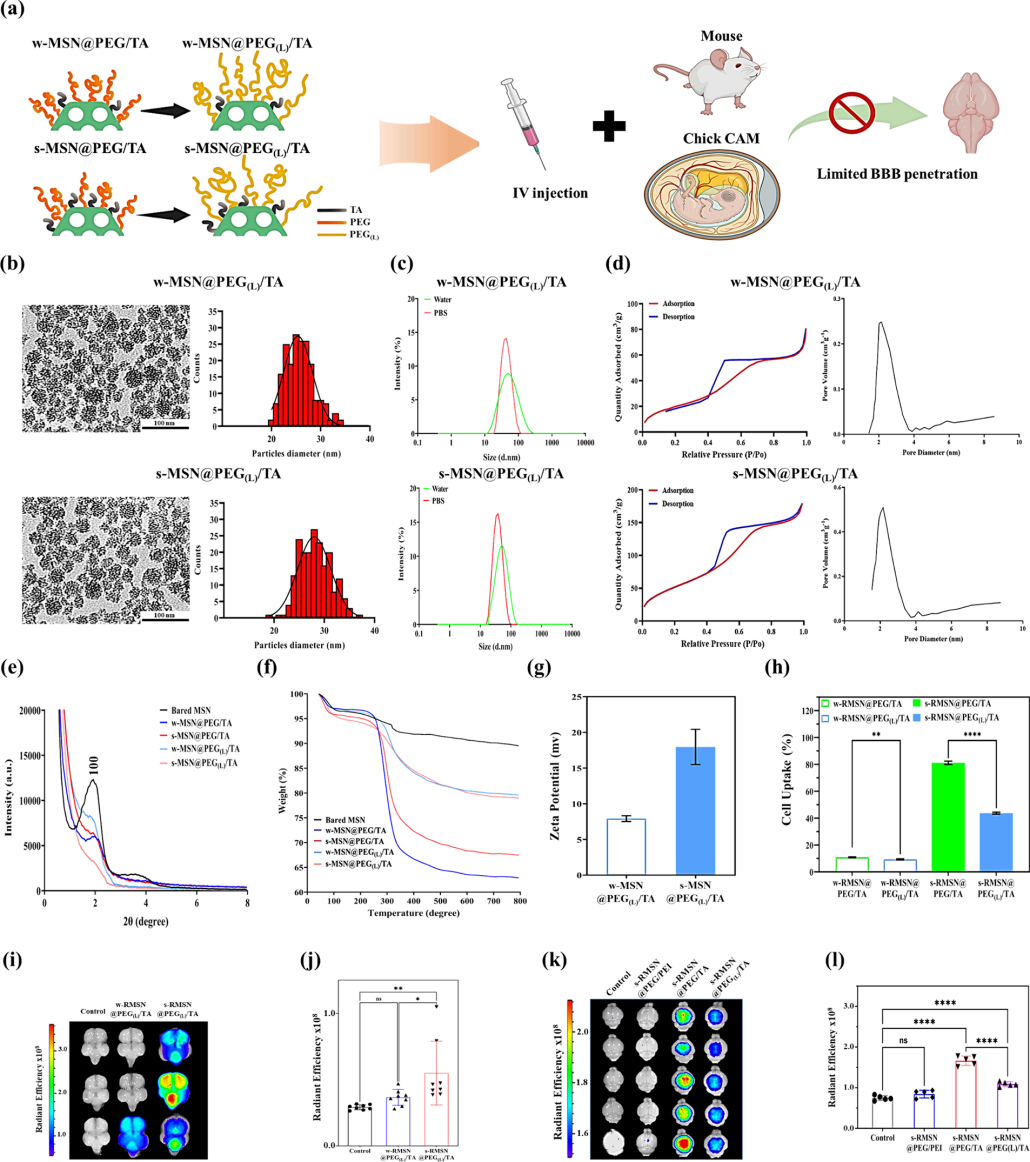
Impact of long-chain PEG on RMSNs for BBB penetration. (a) Schematic illustration of MSN@PEG_(L)_/TA for BBB studies. (b) TEM images and size distribution histograms of MSN@PEG_(L)_/TA. Scale bar: 100 nm. (c) DLS analysis showing the size distribution of MSN@PEG_(L)_/TA in ddH_2_O and PBS. (d) Nitrogen adsorption–desorption isotherms and the corresponding pore size distribution plot of MSN@PEG_(L)_/TA. (e) XRD patterns. (f) TGA-derived weight loss percentages. (g) Zeta potential measurements. (h) Cellular uptake of various RMSNs in U87 cells was assessed by flow cytometry. (i) IVIS imaging and (j) radiant efficiency of RMSN@PEG_(L)_/TA in chick embryonic brains 24 h post-treatment following intravenous injection (1 mg/egg) on embryonic day 15. *N* = 8. (k) IVIS imaging and (l) radiant efficiency of various RMSNs injected intravenously at 200 mg/kg body weight in mouse brains. *N* = 5. Data are presented as mean ± SD. Statistical significance was analyzed using Student’s *t* test (*p < 0.05, **p < 0.01, ***p < 0.001, ****p < 0.0001).

**Table 2:** Basic Characterization of MSN@PEG_(L)_/TA

		**size (nm)** ^a^				
**sample**	**average size from TEM (nm)**	**water (PDI)** ^b^	**PBS (PDI)** ^b^	**ζ-potential (mv)** ^c^	***S***_**BET**_ **(m^2^/g)** ^d^	***D***_**BJH**_ **(nm)** ^e^
w-MSN@PEG_(L)_/TA	25.84 ± 2.96	48.0 ± 0.38 (0.27)	46.6 ± 0.46 (0.25)	7.9 ± 0.41	91.8	2.1
s-MSN@PEG_(L)_/TA	28.22 ± 3.11	53.3 ± 0.42 (0.39)	49.0 ± 0.39 (0.38)	18.0 ± 2.47	210.2	2.2

a Measured by DLS.

b Polydispersity index.

c Measured by a zeta potential analyzer.

d *S*_BET_: surface area calculated from data using the BET equation.

e *D*_BJH_: pore diameter assigned from the maximum on the BJH pore size distribution.

Surface charge measurements revealed that w-MSN@PEG_(L)_/TA and s-MSN@PEG_(L)_/TA had zeta potentials of 7.9 ± 0.41 mV and 18.0 ± 2.47 mV, respectively, which are similar to the zeta potentials of w-MSN@PEG/TA (9.1 ± 1.08 mV) and s-MSN@PEG/TA (18.1 ± 1.10 mV) (Figure 5g and Table 2). Elemental Analysis confirmed the successful conjugation of long-chain PEG onto MSNs, as indicated by detectable carbon content (Table S1). Notably, s-MSN@PEG_(L)_/TA exhibited a higher degree of TA modification, as evidenced by increased nitrogen content compared to w-MSN@PEG_(L)_/TA, resulting in a higher strong positive charge. Overall, the successful preparation of MSN@PEG_(L)_/TA was validated by increased particle size, detectable carbon content, and weight loss patterns. While the surface charge trends of MSN@PEG/TA and MSN@PEG_(L)_/TA were comparable, the primary difference lay in the PEG chain length. Using data from TEM, TGA, XRD, Elemental analysis, and BET pore size measurements, we calculated the PEG density for MSN@PEG/TA and MSN@PEG_(L)_/TA (see Supporting Information for details). Based on the surface area available for PEG modification per gram (nm^2^/g) (Table S3) and the number of PEG molecules (Table 3), the PEG density for w-MSN@PEG/TA was approximately 3.52 chains/nm^2^, while that for s-MSN@PEG/TA was about 1.69 chains/nm^2^. Similarly, the PEG density for w-MSN@PEG_(L)_/TA was 0.42 chains/nm^2^, while that for s-MSN@PEG_(L)_/TA was 0.12 chains/nm^2^. These results indicate significant differences in PEG density among the four materials.

**Table 3:** Comparison of PEG Grafting Density on MSN@PEG/TA and MSN@PEG_(L)_/TA

sample	weight of functionalized MSN^a^ (g)	weight of functional groups^b^ (g)	nitrogen percentage (*N* %) of TA^c^	molecular weight (M.W.) of TA^d^	number of nitrogen atoms in TA^e^	weight of TA^f^ (g)	weight of PEG^g^ (g)	number of PEG molecules^h^ (chains)	PEG density^i^ (chains/nm^2^)	PEG conformation^j^
w-MSN@PEG/TA	1.377	3.774 × 10^–1^	0.386	101.2	1	3.843 × 10^–2^	3.390 × 10^–1^	5.065 × 10^20^	3.52	dense brush
s-MSN@PEG/TA	1.267	2.674 × 10^–1^	0.891	101.2	1	8.162 × 10^–2^	1.858 × 10^–1^	2.777 × 10^20^	1.96	dense brush
w-MSN@PEG_(L)_/TA	1.095	0.953 × 10^–1^	0.710	101.2	1	5.621 × 10^–2^	3.908 × 10^–2^	5.840 × 10^19^	0.42	brush
s-MSN@PEG_(L)_/TA	1.122	1.223 × 10^–1^	1.385	101.2	1	11.24 × 10^–2^	9.982 × 10^–3^	1.492 × 10^19^	0.12	mushroom-brush

a Weight of functionalized MSN $\left(Z\right)=\frac{1}{1-\mathrm{TGA}\;\mathrm{functional}\;\mathrm{group}\;\mathrm{weight}\;\mathrm{percentage}}g$

b Weight of functional groups = Z – 1 g.

c Given from the nitrogen percentage (*N* %) obtained by elemental analysis (Table S1).

d Combustible M.W. of TA-saline: M.W. = 257.83; Effective M.W. used for TGA calculation: M.W. = 101.2.

e Based on the molecular structure of TA (*N* = 1).

f Weight of $\mathrm{TA}=\frac{\frac{Z\times N\%}{14}}{1}\times {\mathrm{TA}}_{M.W.}g$

g Weight of PEG = (Z – 1) – weight of TA g.

h Number of $\mathrm{PEG}\;\mathrm{Molecules}=\frac{\mathrm{Weight}\;\mathrm{of}\;\mathrm{PEG}}{{\mathrm{PEG}}_{M.W.}}\times 6.0221\times 10^{23}$ chains.

i $\mathrm{PEG}\;\mathrm{density}=\frac{\mathrm{Number}\;\mathrm{of}\;\mathrm{PEG}\;\mathrm{molecules}}{S_{\mathrm{PEG}}\mathrm{per}\;\mathrm{gram}}\mathrm{chains}/{\mathrm{nm}}^{2}$

j PEG conformation is referenced in Reference 36, where it is categorized as follows: mushroom (<0.2 chains/nm^2^), brush (0.2–0.5 chains/nm^2^), and dense brush (>0.5 chains/nm^2^).

To evaluate the impact of long-chain PEG on cellular internalization, U87 glial cells were exposed to varying concentrations of RITC-conjugated MSN@PEG_(L)_/TA (RMSN@PEG_(L)_/TA, 20–400 μg/mL), with no significant cytotoxicity observed (Figure S3). Subsequently, U87 glial cells were treated with various RMSN@PEG_(L)_/TA at a concentration of 1000 μg/mL for 24 h, and cellular uptake was analyzed using flow cytometry (Figure 5h). As expected, no significant cellular uptake was observed in the w-RMSN@PEG/TA and w-RMSN@PEG_(L)_/TA-treated groups. However, the cellular uptake of s-RMSN@PEG_(L)_/TA (∼40%) was notably lower than that of s-RMSN@PEG/TA (∼80%), confirming that the long-chain PEG effectively shields TA molecules and reduces cell internalization due to steric hindrance. Subsequently, we investigated the in vivo penetration efficiency of RMSN@PEG_(L)_/TA across the BBB. Chick CAM models with an intact BBB at embryonic day 15 were randomly assigned to three experimental groups (N = 8): control, w-RMSN@PEG_(L)_/TA, and s-RMSN@PEG_(L)_/TA. Following intravenous injection (1 mg/egg), the distribution of NPs in the brain was analyzed 24 h post-treatment using an IVIS imaging system. As shown in Figure 5i, the RITC signals were significantly reduced in both RMSN@PEG_(L)_/TA-treated groups compared to the w-RMSN@PEG/TA-treated groups (Figure 3c), suggesting that the long-chain PEG hinders BBB penetration. Additionally, the radiant efficiency of fluorescence intensity indicated that s-RMSN@PEG_(L)_/TA maintained a stronger signal than w-RMSN@PEG_(L)_/TA, likely due to its enhanced positive charge, which contributed to superior BBB penetration efficacy (Figure 5j).

To verify the chick CAM results, a similar experiment was conducted in mice. Mice were intravenously injected with 200 mg/kg body weight of various RMSNs, focusing on those with stronger positive charges. After 24 h, the distribution of s-RMSN@PEG/PEI, s-RMSN@PEG/TA, and s-RMSN@PEG_(L)_/TA in the brain was visualized using an IVIS imaging system to capture the RITC fluorescence signals. As shown in Figure 5k, detectable fluorescent signals were observed in the s-RMSN@PEG/TA and s-RMSN@PEG_(L)_/TA-treated groups, whereas the s-RMSN@PEG/PEI-treated groups did not show any signal. Notably, the radiant efficiency of fluorescence intensity in the s-RMSN@PEG/TA-treated groups was higher than in the w-RMSN@PEG_(L)_/TA-treated groups (Figure 5l), suggesting that the long-chain PEG effectively shields TA molecules, reducing BBB penetration capability. The results from the mouse model were consistent with those from the chick CAM model, further demonstrating the potential of chick CAM as a viable BBB model. These findings highlight the vital role of the positively charged TA molecule in facilitating MSN transport across the BBB, while also revealing the potential drawbacks of long-chain PEG, which may hinder BBB penetration due to steric hindrance.

## 4 Discussion

Functionalization of MSNs with positively charged molecules is a strategic approach for enhancing drug delivery across the BBB. Tertiary amines, particularly those in the branched cationic polymer polyethyleneimine (PEI), are highly toxic to cells, posing a safety issue.^44^ The positive charge of branched PEI can be conjugated with NPs as carriers to enhance BBB penetration. However, concerns arise due to the inherent high toxicity of tertiary amines, especially when combined with high-molecular-weight PEI, leading to significant disruption of cell membranes and cytotoxicity. To overcome this shortcoming, surface modifications with PEGylation (attaching polyethylene glycol chains) can both enhance NP stability in the bloodstream and mitigate cytotoxicity.^45^ However, achieving a critical balance in optimizing the density and ratio of PEG and PEI on NPs is challenging but crucial for enhancing BBB penetration without compromising safety. Additionally, protein corona (PC) formation, in which serum proteins adsorb onto the NP surface, and buffering capacity can impact the cellular uptake of PEI-NPs, thus affecting NPs biodistribution and BBB penetration.^46^

Alternatively, the quaternization of primary amines to form quaternary ammonium groups has been proposed as a strategy to mitigate toxicity while maintaining the overall positive charge of NPs.^47^ This method produces desirable positively charged NPs that interact favorably with negatively charged cell membranes, thereby facilitating BBB penetration. Our recent findings have proven the potential of MSNs functionalized with a positively charged quaternary ammonium molecule, trimethylammonium (TA), to facilitate BBB penetration in mice.^48^ To further explore the impact of positive charge on BBB crossing, this study delves into the differences in BBB crossing between two types of PEGylated positively charged MSNs (MSN@PEG/PEI and MSN@PEG/TA), each with varying degrees of positive charge, including strong and weak charges. We demonstrated that the quaternary ammonium molecule of TA represents a promising approach, which not only mitigates cytotoxicity but also enhances BBB penetration when functionalized onto RMSNs. In contrast, tertiary amines of branched PEI exhibit significant cytotoxicity and are ineffective at crossing the BBB (Figure 3b and Figure 3c). Although the specific mechanisms associated with BBB penetration by RMSN@PEG/TA remain unclear, we found that its penetration does not involve BBB disruption (Figure 3f). Considering its small size (25 nm) and positive charge, we propose that MSN@PEG/TA may facilitate BBB penetration via diffusion and adsorption-mediated transcytosis mechanisms. To clarify the possible mechanisms, further detailed studies need to be conducted.

As shown in Table S1, element analysis results demonstrated that s-MSN@PEG/TA has a higher TA modification quantity, resulting in enhanced positive charge surface properties. BET pore size analysis (Figure 1e and Table 1) indicates that the pore size of s-MSN@PEG/TA is smaller than w-MSN@PEG/TA, suggesting that the increased TA modification leads to a reduction in pore size, causing lower drug loading. (Figure 4a) Additionally, we employed the positive–negative electrostatic attraction method for drug loading on MSNs. Dox adsorption was facilitated by changing the charge properties through an alkaline solution. Following alkaline treatment at pH 10.0, the surface charge of s-MSN@PEG/TA becomes weakly negative value (−8 mv), while w-MSN@PEG/TA has a stronger negatively charged surface (−17 mv) (Figure S4). Hence, w-MSN@PEG/TA makes it easy for positively charged Dox to adsorb on the MSN surface or pores, whereas the charge properties of s-MSN@PEG/TA make it less favorable for Dox adsorption. Consequently, the drug loading of w-MSN@PEG/TA is higher than that of s-MSN@PEG/TA (Figure 4a). The MSN itself possesses the property of slow drug release. When comparing w-MSN@PEG/TA and s-MSN@PEG/TA, the latter exhibits a slower drug release profile, likely due to its denser TA grafting and smaller pore size, which creates a barrier that impedes drug release (Figure 4b). The cytotoxicity result in Figure 4C was consistent with the drug release results. The slow drug release rate of Dox@s-MSN@PEG/TA leads to lower toxicity compared to Dox@w-MSN@PEG/TA. Additionally, the surface charge of w-MSN@PEG/TA was higher than that of s-MSN@PEG/TA when the MSNs were in either the physiological pH 7.4 or lower pH 5.5 (Figure S4), which enhanced the Dox release due to the charge–charge repulsion.^49,50^ The cytotoxic induction capability of Dox@w-MSN@PEG/TA is higher than Dox@s-MSN@PEG/TA, possibly due to the faster Dox release rate in Dox@w-MSN@PEG/TA, resulting in a higher cumulative release amount (Figure 4b). In this study, cytotoxicity assays were conducted using U87 glioma cells to evaluate the therapeutic relevance of MSN@PEG/TA formulations. While these results provide valuable insights into the efficacy of the nanoparticles, a more comprehensive safety assessment requires further validation in noncancerous human cells. Future studies should include cytotoxicity evaluations in normal cell types, such as human umbilical vein endothelial cells (HUVECs), neuronal stem cells, astrocytes, and potentially in vivo mouse models. Such investigations will be critical for assessing biocompatibility, minimizing off-target toxicity, and advancing the translational potential of MSN-based drug delivery systems for CNS-related therapies.

Biomedical research frequently relies on mice as a model organism due to their physiological and genetic similarities to humans, which have been estimated to be approximately 90%.^51^ These characteristics provide a robust basis for utilizing mice in the pursuit of biomedical research. However, the process of obtaining results using mouse models can be expensive and time-consuming, particularly given the slow progress in drug development for pathological brain diseases.^52^ In addition to mice, zebrafish (Danio rerio) have gained popularity as model organisms, especially for the study of developmental and dynamic processes.^53^ Zebrafish offer advantages such as transparency, genetic manipulability, a high reproductive rate, and the capability for high-throughput screening, making it well suited for studying the BBB.^54^ Despite these benefits, it is essential to acknowledge discrepancies between zebrafish and mammals in terms of anatomy, physiology, and the immune system. As a result, research conducted in zebrafish models often necessitates validation using mouse models. Given these considerations, there is an urgent need to develop an alternative animal model that incorporates the advantages of both mice and zebrafish to achieve highly reproducible results in BBB studies.

The chicken chorioallantoic membrane (CAM) model is a valuable and cost-effective model for conducting biological research, particularly in the areas of angiogenesis, tumor growth, toxicity studies, and drug testing.^35,36^ The CAM model offers a unique balance between fundamental information and detailed research, serving as a bridge between in vitro and in vivo studies.^26^ The benefits of the CAM model^55–58^ include (1) Accessibility: Fertilized chicken eggs are readily available and affordable, making the CAM model accessible to a wide range of researchers; (2) Transparency: The CAM model allows for easy observation of extensive vascularization, tumor growth, and other biological phenomena in real time; (3) In vivo experiments: The model provides an in vivo environment, resulting in observations that are more relevant to human biology compared to in vitro models; (4) Short experimental duration: Rapid development of the CAM model often shortens experimental times compared to other mammalian animal models; (5) Natural immunodeficiency: In the early stages of development, the CAM model lacks a well-developed immune system, which is beneficial for the growth of implanted xenogeneic tumor cells and the formation of vascular networks; (6) Ethical Advantages: Given the absence of pain perception, the chick CAM model is not considered a living animal until E17 (in most countries) or even until hatching. Experiments involving chick CAM do not require ethical restrictions or protocol approval from animal welfare or ethics committees, thus simplifying the planning process. In this study, we have proven that the chick CAM model provides a good BBB penetration evaluation platform with comparable outcomes with the mouse model. With a lower cost in comparison to mouse experiments and a closer in vivo environment when compared to zebrafish, the chick CAM model could be an alternative animal model for biomedical applications.

Evidence suggests that the molecular weight (MW) and density of PEG are critical factors influencing the physicochemical and biological properties of NPs.^59–61^ In this study, we focus on the impact of PEG chain length on BBB penetration when conjugated onto the NP surface. PEGylation, the process of conjugating PEG onto NPs, creates a hydrophilic and flexible surface that enhances NP circulation in the bloodstream, reduces clearance by the mononuclear phagocyte system (MPS), and minimizes protein corona formation.^62^ While PEGylation, particularly with long-chain PEG, provides these benefits, it also presents challenges, especially in terms of its effects on the biomolecular function and behavior of NPs. The main issues include (1) Reduced Cellular Uptake: the hydrophilic nature of long-chain PEG reduces interactions between NPs and the largely hydrophobic cell membranes, significantly decreasing cellular uptake. This limits the intracellular delivery of therapeutic agents;^61^ (2) Steric Hindrance: excessively long PEG chains can create steric hindrance around the NP surface, hindering the binding efficiency of targeted ligands, such as antibodies or aptamers. This steric barrier of the PEG layer may prevent these ligands from effectively interacting with their targets, diminishing targeting efficiency.^63,64^ In our study, we observed that long-chain PEG effectively shields TA molecules, leading to reduced cell internalization in U87 cells (Figure 5d) and impaired BBB penetration in vivo (Figure 5e and Figure 5g) due to steric hindrance. This finding underscores the potential impact of PEG chain length on the effectiveness of NPs in crossing the BBB. We found that TA was the key element that facilitated the BBB crossing of the MSNs.

Densely packed PEG chains can affect the formation of the protein corona and mask targeting ligands on the NP surface, particularly when NPs are intended to cross the BBB.^65^ The protein corona alters the behavior and fate of NPs and may also obscure surface-bound targeting ligands. NPs coated with PEG in a low-density mushroom configuration offer weak resistance to serum protein adsorption due to the creation of a thinner hydrophobic barrier.^66^ In contrast, a dense brush configuration of PEG forms a thicker hydrophilic barrier that is more effective at blocking protein adsorption.^67^ Walkey et al. reported that steric hindrance from the high grafting density of PEG chains in the brush configuration could limit serum protein binding.^67^ However, even at high PEG density, complete prevention of protein adsorption is not achievable, and excessive PEGylation may also inhibit cellular uptake.^68,69^ Therefore, fine-tuning the MW and surface density of PEG, along with the biophysical and chemical characteristics of NPs, is critical for improving BBB penetration and optimizing drug delivery to the brain. To address these challenges, it is essential to carefully design the PEGylation process to balance stealth properties and functional efficacy. A common strategy to reduce the shielding effect is to optimize the length or density of PEG chains. In this study, we used short-PEG chains (M.W. 459–591) and incorporated TA at two different PEG-to-TA ratios. As shown in Table 2, we clearly verified that both w-MSN@PEG/TA (PEG density: 3.52 chains/nm^2^) and s-MSN@PEG/TA (PEG density: 1.96 chains/nm^2^) exhibited a brush-like configuration of PEG coating, consistent with previous reports.^65,70^ We demonstrated that optimizing short PEG density in MSNs design not only retains the inherent benefits of PEG for in vivo applications but also preserves the functionality of TA, thereby facilitating effective BBB crossing. Given that short PEG chains enhance compatibility, our data show that RMSN@PEG/TA exhibited excellent BBB penetration without causing damage to the barrier (Figure 3c and Figure 3f). Moreover, compared to s-RMSN@PEG_(L)_/TA (PEG: M.W. 1k), s-RMSN@PEG/TA demonstrated superior efficacy in crossing the BBB, highlighting the advantages of the short PEG design in mice (Figure 5k and Figure 5l). Overall, the small size, short-PEG/TA modification, and strong positive charge of s-RMSN@PEG/TA contributed to its ability to cross the BBB.

## 5 Conclusions

In this study, we synthesized MSNs with controllable size, charge, and PEG length, focusing on their ability to penetrate the BBB. Surface modification with the positively charged quaternary ammonium molecule TA, along with optimized short PEG density, is crucial for enabling BBB penetration of MSNs. MSN@PEG/TA was found to have a great BBB penetration ability without causing BBB damage. By systematically exploring the BBB penetration capabilities of MSN@PEG/PEI and MSN@PEG/TA, this understanding is anticipated to contribute valuable information for the development of safe and effective drug delivery systems that target the BBB. Additionally, we established the chick CAM as a BBB model, which served as a versatile and accessible in vivo model due to its natural advantages. Its applications span across angiogenesis, tumor growth, toxicity studies, and drug testing, making it a valuable tool in biological research, particularly in the context of cancer- and angiogenesis-related studies. Here, we successfully demonstrated that the chick CAM model offers refined methodologies for studying the BBB, which may contribute to the broader perspective of future brain-related research with the value of translational medicine.

## Supplementary Material



## Data Availability

Data will be made available on request.

## Notes

Elemental analysis, fluorescence images, 3D confocal microscopy images, cytotoxicity, zeta potential, FTIR spectroscopy, TGA analysis, TEM, and BET (PDF)
