# Supplementary material for: Tailored Mesoporous Silica Nanoparticles and the Chick Chorioallantoic Membrane: A Promising Strategy and Model for Efficient Blood-Brain Barrier Crossing
Source: ACS Appl Mater Interfaces. 2025 May 6;17(20):29437–54. doi: 10.1021/acsami.5c05429 (PMC12100600; doi:10.1021/acsami.5c05429)
Supplement: Supplementary file 1 [file am5c05429_si_001.pdf]

Supporting Information

Tailored Mesoporous Silica Nanoparticles and the Chick Chorioallantoic Membrane: A Promising Strategy and Model for Efficient Blood-Brain Barrier Crossing

Cong-Kai Lin[a†], Yi-Shan Yang[b†], Tsang-Pai Liu[c], Jiunn-Chang Lin[c,d], Sasinan Bupphathong[e], Fuyuhiko Tamanoi[f] and Yi-Ping Chen[e,g]*

[a]Graduate Institute of Biomedical Materials Tissue Engineering, College of Biomedical Engineering, Taipei Medical University, Taipei 110, Taiwan

[b]Department of Neurosurgery, Taipei Medical University Hospital, Taipei 110, Taiwan

[c]Department of Surgery, MacKay Memorial Hospital, Taipei 104, Taiwan

[d]MacKay Junior College of Medicine, Nursing and Management, New Taipei 252, Taiwan

[e]Graduate Institute of Nanomedicine and Medical Engineering, College of Biomedical Engineering, Taipei Medical University, Taipei 110, Taiwan

[f]Institute for Integrated Cell-Material Sciences, Institute for Advanced Study, Kyoto University, Kyoto 606-8501, Japan

[g]International Ph.D. Program in Biomedical Engineering, College of Biomedical Engineering, Taipei Medical University, Taipei 110, Taiwan

[†] These authors contributed equally to this work

*** Correspondence: haychen@tmu.edu.tw**

Table S1.

The percentage of carbon (C), hydrogen (H), and nitrogen (N) content for the MSNs obtained from elemental analysis.

Sample	C%	H%	N%
**Bare MSN**	2.755	2.050	0.090
**w-MSN@PEG/TA**	17.949	4.096	0.476
**s-MSN@PEG/TA**	15.571	3.645	0.981
**w-MSN@PEG/PEI**	24.057	4.710	0.197
**s-MSN@PEG/PEI**	23.385	4.442	0.277
**w-MSN@PEG$_{(L)}$/TA**	13.030	3.665	0.730
**s-MSN@PEG$_{(L)}$/TA**	11.780	3.561	1.100

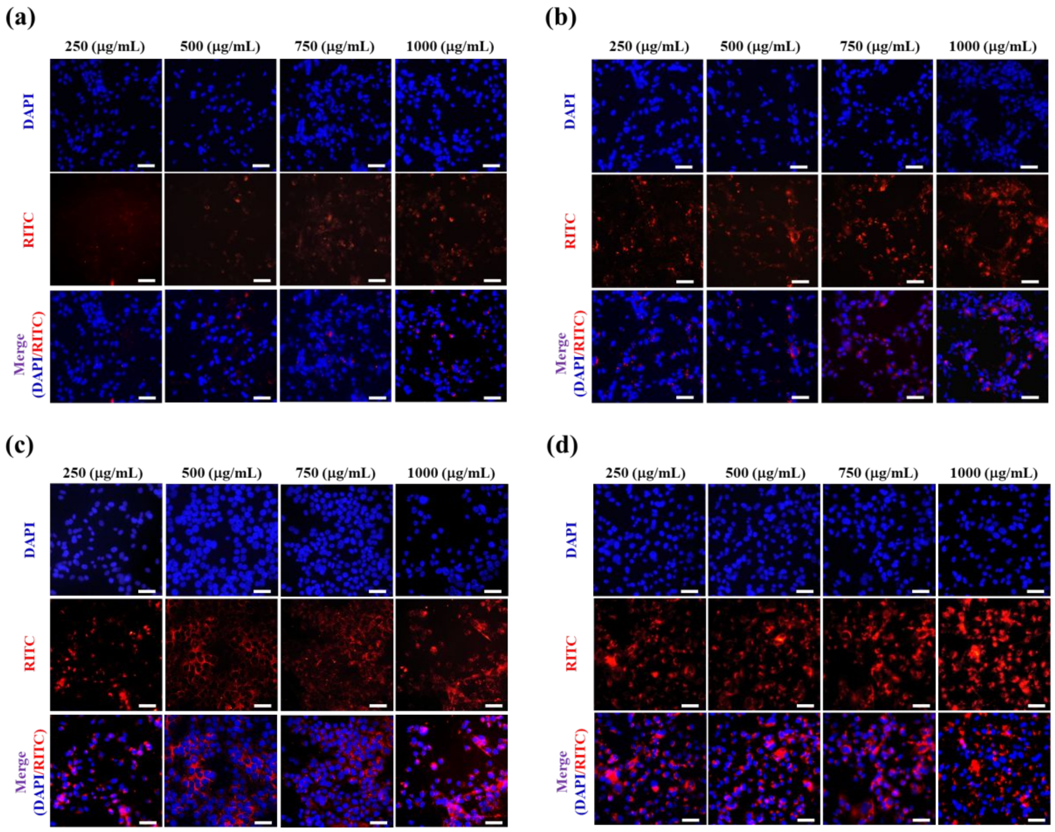

Figure S1. Fluorescence images showing cellular uptake of RMSNs at various concentrations (a) w-RMSN@PEG/TA, (b) s-RMSN@PEG/TA, (c) w-RMSN@PEG/PEI, and (d) s-RMSN@PEG/PEI. (Red: RMSN, Blue: DAPI for nuclei). Scale bar: 50 μm.

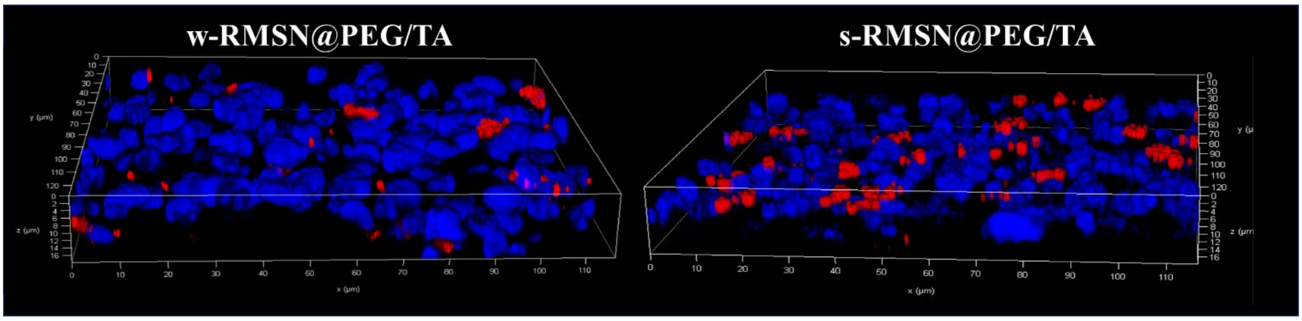

Figure S2. 3D reconstructed confocal microscopy images of brain sections. (Red: RMSN@PEG/TA; Blue: DAPI-stained nuclei).

Table S2.

Thermal stability and weight loss of various MSNs based on TGA.

Samples	TGA results for 40°C-200°C (wt %)	TGA results for 200°C-600°C (wt %)	TGA results for 600°C-800°C (wt %)
**Bare MSN**	3.9%	5.3%	1.2%
**w-MSN@PEG/TA**	3.3%	33.2%	0.7%
**s-MSN@PEG/TA**	4.9%	26.7%	0.9%
**w-MSN@PEG$_{(L)}$/TA**	1.4%	14.4%	0.8%
**s-MSN@PEG$_{(L)}$/TA**	2.6%	16.0%	1.4%

Wt %: normalized weight loss from thermogravimetric analysis (TGA) analysis.

Table S3.

Properties of various MSNs.

Sample	Diameter[a] (nm)	Radius (r, nm)	S_{tot} per MSN[b] (cm^2)	V per MSN[c] (cm^3)	Number of channels per MSN[d]	A_{holes}[e] (cm^2)	S_{PEG} per MSN[f] (cm^2)	Porosity[g]	Weight of MSN[h] (g)	Number of MSN per[i] gram	S_{PEG} per gram[j] (nm^2/g)
**Bare MSN**	25.2	12.6	2.993 x 10^{-11}	1.257 x 10^{-17}	2.543 x 10^{1}	3.132 x 10^{-12}	2.679 x 10^{-11}	3.139 x 10^{-1}	1.897 x 10^{-17}	5.271 x 10^{16}	1.412x 10^{20}
**w-MSN@PEG/TA**	24.7	12.4	2.875 x 10^{-11}	1.184 x 10^{-17}	2.443 x 10^{1}	3.009 x 10^{-12}	2.574 x 10^{-11}	3.139 x 10^{-1}	1.786 x 10^{-17}	5.598 x 10^{16}	1.441 x 10^{20}
**s-MSN@PEG/TA**	25.1	12.6	2.969 x 10^{-11}	1.242 x 10^{-17}	2.523 x 10^{1}	3.107 x 10^{-12}	2.658 x 10^{-11}	3.139 x 10^{-1}	1.875 x 10^{-17}	5.335 x 10^{16}	1.418 x 10^{20}
**w-MSN@PEG$_{(L)}$/TA**	25.8	12.9	3.137 x 10^{-11}	1.349 x 10^{-17}	2.665 x 10^{1}	3.282 x 10^{-12}	2.809 x 10^{-11}	3.139 x 10^{-1}	2.036 x 10^{-17}	4.912 x 10^{16}	1.380 x 10^{20}
**s-MSN@PEG$_{(L)}$/TA**	28.2	14.1	3.747 x 10^{-11}	1.761 x 10^{-17}	3.184 x 10^{1}	3.922 x 10^{-12}	3.355 x 10^{-11}	3.139 x 10^{-1}	2.658 x 10^{-17}	3.762 x 10^{16}	1.262 x 10^{20}

a. The diameter of MSNs obtained from TEM average diameter measurements is provide in Table 1.
b. S_{tot} **per MSN =** $6 \times \pi \times r^2 \times 10^{-14}\ cm^2$.
c. V per MSN = $2 \times \pi \times r^3 \times 10^{-21}\ cm^3$.
d. Number of channels per MSN = $\left(\frac{\pi \times r^2}{\frac{3}{2} \times \sqrt[2]{3} \times a^2}\right) \times 3$.
e. A_{holes} = Number of channels per MSN $\times 2 \times \pi \times \left(\frac{\text{Pore diameter from bare MSN}}{2} \times 10^{-7}\right)^2\ cm^2$.
f. S_{PEG} **per MSN =** $S_{tot} - A_{holes}\ cm^2$.
g. Porosity = $\frac{\text{Pore Volume}}{\text{Cylinder } Volume} = \frac{\text{Number of channels per MSN} \times \left(\frac{\text{Pore diameter from bare } MSN}{2}\right)^2}{(\text{Diameter})^2}$.
h. Weight of MSN = Volume × Density of Silica × (1 − Porosity) g.
i. Number of MSNs per gram = $\frac{1\ g}{\text{Weight of MSN g}}$.
j. S_{PEG} **per gram =** S_{PEG} **per MSN × Number of MSNs per gram ×** $10^{14}\ nm^2/g$.

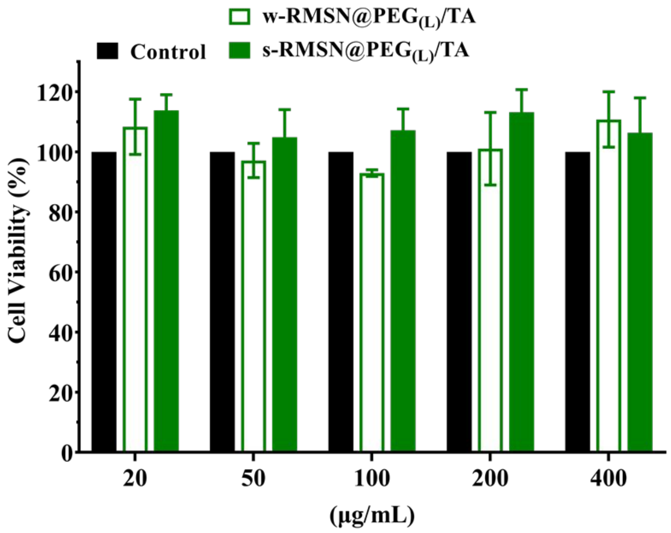

Figure S3. Cytotoxicity of RMSN@PEG$_{(L)}$/TA in U87 cells. U87 cells were treated with RMSN@PEG$_{(L)}$/TA at various concentrations. Cytotoxicity was assessed using the CCK-8 assay.

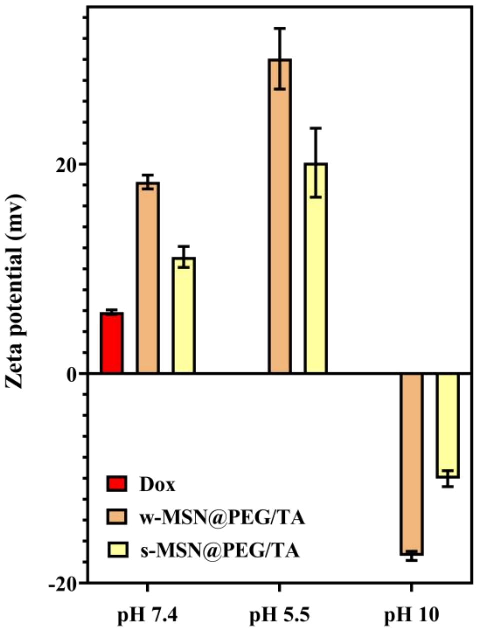

Figure S4. Zeta potential of various MSNs at pH 5.5, 7.4, and 10 and of Dox at pH 7.4.

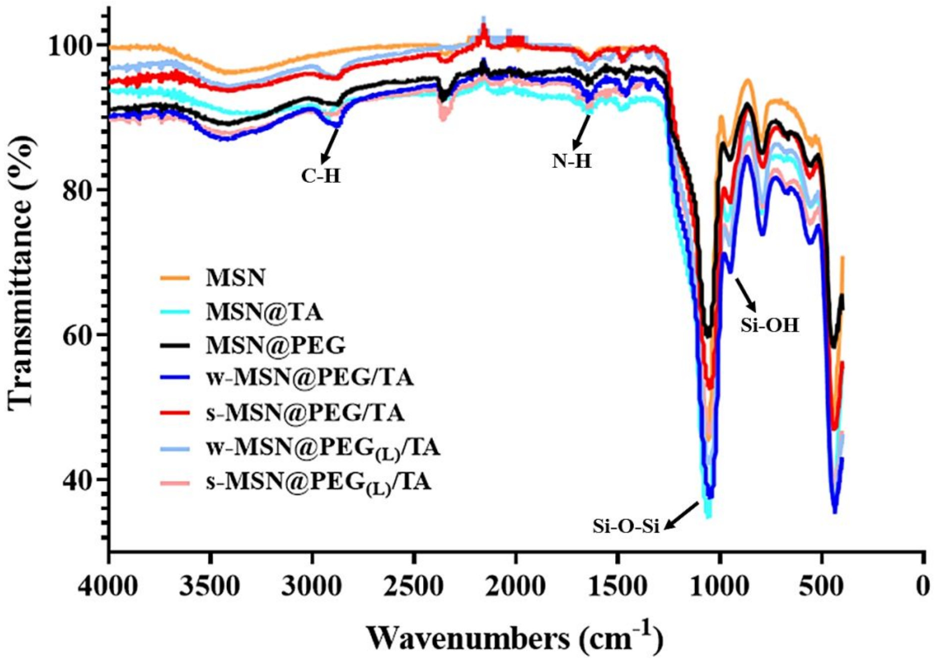

Figure S5. FTIR spectroscopy confirmed the successful surface modification of MSNs with PEG- and TA-silane. Characteristic peaks were observed at ~2880 cm⁻¹ (C–H stretching from PEG), 1000–1200 cm⁻¹ (Si–O–Si stretching), and ~950 cm⁻¹ (Si–OH bending). All TA-modified MSNs showed enhanced absorption at 1550–1650 cm⁻¹, corresponding to the N–H stretching of quaternary ammonium groups.

Assumptions and Given Data

The known data:

1. **Shape**:

   The basic parameters of bare MSN obtained from TEM (for particle diameter) and BET (for pore diameter) are as follows:

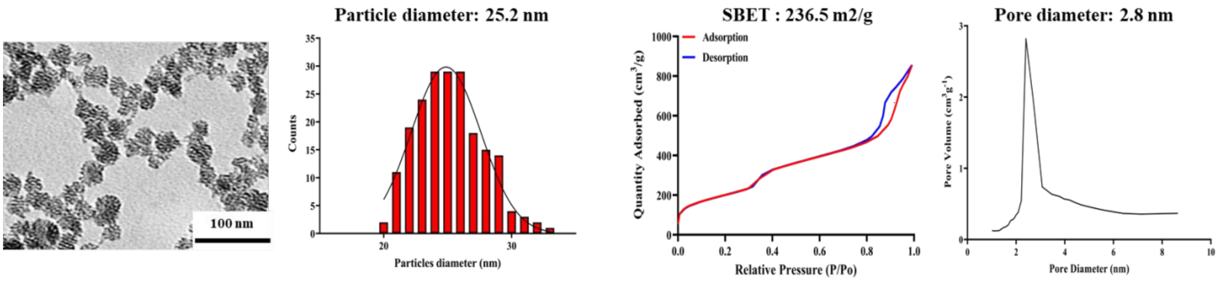

   Bare MSN is assumed to have a cylindrical structure with a diameter 2r = 25.2 nm, radius r = 12.6 nm, and a pore diameter of 2.8 nm.

   w-MSN@PEG/TA is assumed to retain a cylindrical structure with a diameter 2r = 24.7 nm and radius r = 12.4 nm (from Table 1).

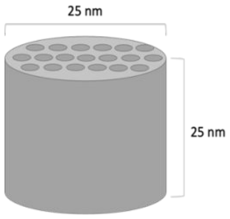

2. **Total outer surface area (S_{tot}) per MSN**:

$$S_{tot} = 6 \times \pi \times r^2 \times 10^{-14} \text{ cm}^2$$

   Bare MSN: $S_{tot} = 6 \times \pi \times (12.6)^2 \times 10^{-14} = 2.993 \times 10^{-11}$ cm^2

   w-MSN@PEG/TA: $S_{tot} = 6 \times \pi \times (12.4)^2 \times 10^{-14} = 2.2.875 \times 10^{-11}$ cm^2

3. **Total volume (V) per MSN**:

$$V = 2 \times \pi \times r^3 \times 10^{-21} \text{ cm}^3$$

   Bare MSN: $V = 2 \times \pi \times (12.6)^3 \times 10^{-21}$ cm^3 $= 1.257 \times 10^{-17}$ cm^3

   w-MSN@PEG/TA: $V = 2 \times \pi \times (12.4)^3 \times 10^{-21}$ cm^3 $= 1.184 \times 10^{-17}$ cm^3

XRD data of bare MSN

From the XRD data (Figure 5E), the diffraction angle is determined as 2θ = 2.142. (h, k, l=1, 0, 0)

Using Bragg's Law:

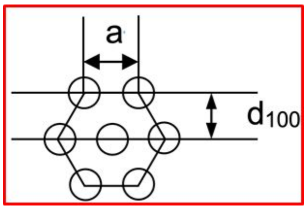

$$d_{100} = \frac{\lambda}{2\sin\theta} = \frac{1.5406\times10^{-10}}{2\sin(\frac{2.142}{2})\times10^{9}} = 4.121 \text{ nm}$$

and the lattice constant (a):

$$a = \left(\frac{1}{(d_{hkl})^2}\right) = \frac{4}{3}\left(\frac{h^2+hk+k^2}{a^2}\right) = \sqrt{\frac{4}{3}} \times d_{100} = 1.1547 \times 4.121 = 4.759\ nm$$

Given d_{100} = 4.121 nm and the lattice constant (a) = 4.759 nm, the structural properties are calculated accordingly.

Calculations

Examples for Bare MSN and w-MSN@PEG/TA:

1. Assumptions:

(i) MSNs exhibit a hexagonal disk shape with an average particle size and thickness of 25 nm by TEM.

(ii) The pores are unidirectionally aligned along the c-axis, as observed through XRD.

(iii) The contribution of the outer surface was not considered in this analysis.

2. Number of pores per hexagonal unit (shown in red color):

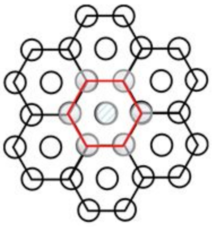

$$1_{\text{complete hexagon}} + \left(\frac{1}{3}_{\text{hexagon}}\right) \times 6 = 3_{\text{complete hexagon}}$$

3. Number of channels per MSN:

$$\text{Number of channels per MSN} = \left(\frac{\pi \times r^2}{\frac{3}{2} \times \sqrt[2]{3} \times a^2}\right) \times 3$$

Bare MSN: Number of channels MSN = $\left(\frac{\pi\times(12.6)^2}{\frac{3}{2}\times\sqrt[2]{3}\times4.759^2}\right) \times 3 = 25.43$

w-MSN@PEG/TA: Number of channels MSN = $\left(\frac{\pi\times(12.4)^2}{\frac{3}{2}\times\sqrt[2]{3}\times4.759^2}\right) \times 3 = 24.43$

Based on the hexagonal unit and arrangement, the calculated number of channels for bare MSN and w-MSN@PEG/TA is 25.43 and 24.43, respectively.

4. Total Surface area of holes on two circular bases (A_{holes}):

$$A_{holes} = \text{Number of channels per MSN} \times \text{Hole area per channel}$$

$$A_{holes} = \text{Number of channels per MSN} \times 2 \times \pi \times \left(\frac{\text{Pore diameter from bare MSN}}{2} \times 10^{-7}\right)^2 \text{cm}^2$$

Bare MSN: $A_{holes} = 25.43 \times 2 \times \pi \times \left(\frac{2.8}{2} \times 10^{-7}\right)^2 = 3.132 \times 10^{-12}$ cm^2

w-MSN@PEG/TA: $A_{holes} = 24.43 \times 2 \times \pi \times \left(\frac{2.8}{2} \times 10^{-7}\right)^2 = 3.009 \times 10^{-12}$ cm^2

3. Available surface area for PEG modification (S_{PEG}) per MSN:

$$S_{PEG} = S_{tot} - A_{holes} \text{ cm}^2$$

Bare MSN: $S_{PEG} = S_{tot} - A_{holes} = (2.993 \times 10^{-11}) - (3.132 \times 10^{-12}) = 2.679 \times 10^{-11}$ cm^2

w-MSN@PEG/TA: $S_{PEG} = S_{tot} - A_{holes} = (2.875 \times 10^{-11}) - (3.009 \times 10^{-12}) = 2.574 \times 10^{-11}$ cm^2

4. Porosity: fixed value calculated from bare MSN

$$\text{Porosity} = \frac{\text{Pore Volume}}{\text{Cylinder Volume}} = \frac{\text{Number of channels per MSN} \times \left(\frac{\text{Pore diameter from bare MSN}}{2}\right)^2}{(\text{Diameter})^2}$$

Bare **MSN** = $\left(\frac{25.43 \times \left(\frac{2.8}{2}\right)^2}{\left(\frac{25.2}{2}\right)^2}\right) = 3.139 \times 10^{-1}$

5. Weight of MSN:

$$\text{Weightof MSN} = \text{Volume} \times \text{Density of Silica} \times (1 - \text{Porosity})$$

The density of silica is 2.2 g/cm^3

Bare MSN: $(1.257 \times 10^{-17}) \times (2.2) \times (1 - 3.139 \times 10^{-1}) = 1.897 \times 10^{-17}$ g

w-MSN@PEG/TA: $(1.184 \times 10^{-17}) \times (2.2) \times (1 - 3.139 \times 10^{-1}) = 1.786 \times 10^{-17}$ g

6. Number of MSN per Gram:

$$\text{Number of MSN per Gram} = \frac{1 \text{ g}}{\text{Weight of MSN g}}$$

Bare MSN $= \frac{1 \text{ g}}{1.897 \times 10^{-17} \text{g}} = 5.271 \times 10^{16}$

w-MSN@PEG/TA $= \frac{1 \text{ g}}{1.786 \times 10^{-17} \text{g}} = 5.598 \times 10^{16}$

7. Available surface area for PEG modification (S_{PEG}) per Gram:

$$\text{Area} = S_{PEG} \times \text{Number of MSN per Gram} \times 10^{14} \text{ nm}^2/\text{g}$$

Bare MSN = $(2.679 \times 10^{-11}) \times (5.271 \times 10^{16}) \times 10^{14} = 1.412 \times 10^{20}$ nm^2/g

w-MSN@PEG/TA = $(2.574 \times 10^{-11}) \times (5.598 \times 10^{16}) \times 10^{14} = 1.441 \times 10^{20}$ nm^2/g

Steps for Calculating PEG Density Using TGA and EA Data

1. Calculate the weight of functionalized MSN:

$$\text{Weight of functionalized MSN (Z)} = \frac{1}{1-\text{TGA functional group weight percentage}}$$

w-MSN@PEG/TA: $Z = \frac{1}{1-27.4\%} = 1.377\ \text{g}$

2. Determine the total weight of functional groups (PEG/TA):

Weight of functional groups = $Z - 1$ g

w-MSN@PEG/TA: $Z - 1 = 1.377 - 1 = 0.377\ \text{g}$

3. Use EA data to determine the nitrogen percentage (*N* %) of TA:

Given from the nitrogen percentage (*N* %) obtained by elemental analysis (Table S1).

w-MSN@PEG/TA: *N* % = 0.386 %

4. Calculate the molecular weight (M.W.) of TA:

Relevant chemical information to estimate the molecular weight of TA.

Combustible M.W. of TA-saline: M.W.= 257.83

Effective M.W. used for TGA calculation: M.W.=101.2

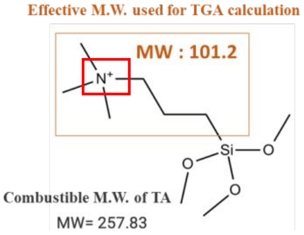

5. Determine the number of nitrogen atoms in TA (shown in red color):

Identify the number of nitrogen atoms per molecule based on the molecular structure of TA.

Number of nitrogen atoms (*N*)=1

6. Calculate the weight of TA using TGA :

$$\text{Weight of TA} = \frac{\frac{Z\times N\%}{14}}{1} \times \text{TA}_{\text{M.W.}}\ \text{g}$$

w-MSN@PEG/TA: Weight of TA = $\frac{\frac{1.377\times 0.386\%}{14}}{1} \times 101.2 = 3.843 \times 10^{-2}\ \text{g}$

7. Calculate the Weight of PEG using TGA:

$$\text{Weight of PEG} = (Z-1) - \text{Weight of TA}\ \text{g}$$

w-MSN@PEG/TA: Weight of PEG = $0.377 - 3.843 \times 10^{-2} = 3.390 \times 10^{-1}\ \text{g}$

8. Calculate the number of PEG molecules (chains):

$$\text{Nnumber of PEG molecules} = \frac{\text{Weight of PEG}}{\text{PEG}_{\text{M.W.}}} \times 6.0221 \times 10^{23}\ \text{chains}$$

w-MSN@PEG/TA: $\text{Number of PEG molecules} = \frac{3.390 \times 10^{-1}}{403} \times 6.0221 \times 10^{23} = 5.065 \times 10^{20}$ chains

9. Calculate PEG density:

$$\text{PEG density} = \frac{\text{Number of PEG molecules}}{S_{PEG}\ \text{per gram}} \quad \text{chains/nm}^2$$

w-MSN@PEG/TA: $\text{PEG density} = \frac{5.065 \times 10^{20}}{1.412 \times 10^{20}} = 3.52$ chains/nm^2

References:

[1] Tu, Hsiung Lin, et al. "In vitro studies of functionalized mesoporous silica nanoparticles for photodynamic therapy." Advanced Materials 21.2 (2009): 172-177.

[2] Pan, Limin, et al. "Nuclear-targeted drug delivery of TAT peptide-conjugated monodisperse mesoporous silica nanoparticles." Journal of the American Chemical Society 134.13 (2012): 5722-5725.

[3] Lin, Yu-Shen, and Christy L. Haynes. "Impacts of mesoporous silica nanoparticle size, pore ordering, and pore integrity on hemolytic activity." Journal of the American Chemical Society 132.13 (2010): 4834-4842.
